# Systematic longitudinal survey of invasive *Escherichia coli* in England demonstrates a stable population structure only transiently disturbed by the emergence of ST131

**DOI:** 10.1101/gr.216606.116

**Published:** 2017-08

**Authors:** Teemu Kallonen, Hayley J. Brodrick, Simon R. Harris, Jukka Corander, Nicholas M. Brown, Veronique Martin, Sharon J. Peacock, Julian Parkhill

**Affiliations:** 1 Wellcome Trust Sanger Institute, Hinxton, Cambridge CB10 1SA, United Kingdom;; 2 Department of Medicine, University of Cambridge, Cambridge CB2 0QQ, United Kingdom;; 3 Department of Mathematics and Statistics, University of Helsinki, 00014 Helsinki, Finland;; 4 Department of Biostatistics, University of Oslo, 0372 Oslo, Norway;; 5 Public Health England, Clinical Microbiology and Public Health Laboratory, Addenbrooke's Hospital, Cambridge CB2 0QQ, United Kingdom;; 6 Cambridge University Hospitals NHS Foundation Trust, Cambridge CB2 0QQ, United Kingdom;; 7 British Society of Antimicrobial Chemotherapy, Birmingham B1 3NJ, United Kingdom;; 8 London School of Hygiene and Tropical Medicine, London WC1E 7HT, United Kingdom

## Abstract

*Escherichia coli* associated with urinary tract infections and bacteremia has been intensively investigated, including recent work focusing on the virulent, globally disseminated, multidrug-resistant lineage ST131. To contextualize ST131 within the broader *E. coli* population associated with disease, we used genomics to analyze a systematic 11-yr hospital-based survey of *E. coli* associated with bacteremia using isolates collected from across England by the British Society for Antimicrobial Chemotherapy and from the Cambridge University Hospitals NHS Foundation Trust. Population dynamics analysis of the most successful lineages identified the emergence of ST131 and ST69 and their establishment as two of the five most common lineages along with ST73, ST95, and ST12. The most frequently identified lineage was ST73. Compared to ST131, ST73 was susceptible to most antibiotics, indicating that multidrug resistance was not the dominant reason for prevalence of *E. coli* lineages in this population*.* Temporal phylogenetic analysis of the emergence of ST69 and ST131 identified differences in the dynamics of emergence and showed that expansion of ST131 in this population was not driven by sequential emergence of increasingly resistant subclades. We showed that over time, the *E. coli* population was only transiently disturbed by the introduction of new lineages before a new equilibrium was rapidly achieved. Together, these findings suggest that the frequency of *E. coli* lineages in invasive disease is driven by negative frequency-dependent selection occurring outside of the hospital, most probably in the commensal niche, and that drug resistance is not a primary determinant of success in this niche.

*Escherichia coli* is a common commensal of the gastrointestinal tract of humans and other vertebrates and can be isolated from soil and water. *E. coli* is also the leading cause of bloodstream infection in England, elsewhere in Europe and the United States (US) (Elixhauser et al. 2011; de Kraker et al. 2013; Gerver et al. 2015). Annual rates increased in England by 80% between 2003 and 2011 (from 16,542 to 29,777), which led to the introduction of mandatory surveillance from 2011. This documented a 10% increase between 2012/2013 and 2014/2015 from 32,309 to 35,676 cases (Gerver et al. 2015). The most common underlying causes for bloodstream infection in a national collection of the British Society for Antimicrobial Chemotherapy (BSAC) Bacteraemia Resistance Surveillance Programme during 2001–2010 related to urinary tract infection (UTI) and gastrointestinal and hepatobiliary infections (Day et al. 2016).

Previous genetic studies of *E. coli* lineages associated with UTI and/or bacteremia in England and the US have reported that the most prevalent multilocus sequence types (MLSTs) are sequence types (STs) ST73, ST131, ST95, and ST69 (Gibreel et al. 2012; Adams-Sapper et al. 2013; Alhashash et al. 2013; Banerjee et al. 2013; Horner et al. 2014). ST131 has received particular attention, following its apparent emergence in the 2000s, due to its rapid global dissemination and frequent multidrug-resistant (MDR) phenotype (Nicolas-Chanoine et al. 2014). This has led to ST131 being well characterized by publications that propose biological explanations for its emergence and spread (Price et al. 2013; Petty et al. 2014; Salipante et al. 2015; Ben Zakour et al. 2016; Stoesser et al. 2016). Other common STs are less well characterized despite their association with disease, in part because they are less often defined as MDR and because ST131 is an important player in the broader global problem of increasing antibiotic resistance in Gram-negative bacteria, with clinical isolates that are resistant to aminoglycosides, fluoroquinolones, extended-spectrum beta-lactamases, carbapenems, and colistin beginning to emerge (Chen et al. 2014; Zhang et al. 2014; Liu et al. 2016; Skov and Monnet 2016).

Many of the published whole-genome sequencing (WGS) studies on *E. coli* have largely concentrated on ST131, with fewer focused on other extraintestinal pathogenic *E. coli* (ExPEC). Studies have characterized ST131 in detail and highlighted genetic events leading to the success of this lineage. Two studies investigating the origin of enteropathogenic *E. coli* (EPEC) and atypical enteropathogenic *E. coli* (aEPEC) and the association of genetic factors with clinical disease severity illustrated the power of WGS by showing that aEPEC and EPEC emerged several times in different lineages (Hazen et al. 2016; Ingle et al. 2016), and a further study analyzed a global collection of 362 enterotoxigenic *E. coli* (ETEC) (von Mentzer et al. 2014). Smaller studies of local epidemics have concentrated on other pathotypes and single STs.

Here, we used WGS to analyze the genetic diversity of a large collection of *E. coli* isolates associated with bloodstream infection over more than a decade, using nested systematic surveys of England and the Cambridge area. These were not selected based on ST or other bacterial characteristics. We investigated trends in population structure and mechanisms of antibiotic resistance and captured the introduction of ST131 and ST69, which enabled us to study the dynamics of emergence and its effect on the wider *E. coli* population.

## Results

### Study design and bacterial isolates

We conducted a retrospective study in which we analyzed WGS data for 1509 *E. coli* isolates drawn from national BSAC (*n* = 1094) and local (*n* = 415) collections. The BSAC collection consisted of isolates submitted to a Bacteraemia Resistance Surveillance Programme (www.bsacsurv.org) between 2001–2011 by 11 hospitals across England. From each hospital, the first 10 isolates (when available) for each year were included into the study. The local collection was sourced from the diagnostic laboratory at the Cambridge University Hospitals NHS Foundation Trust (CUH), Cambridge. By using the laboratory database, we selected every third isolate associated with bacteremia that had been stored in the −80°C freezer archive between 2006 and 2012.

### Phylogeny and pan-genome

**Figure 1.**
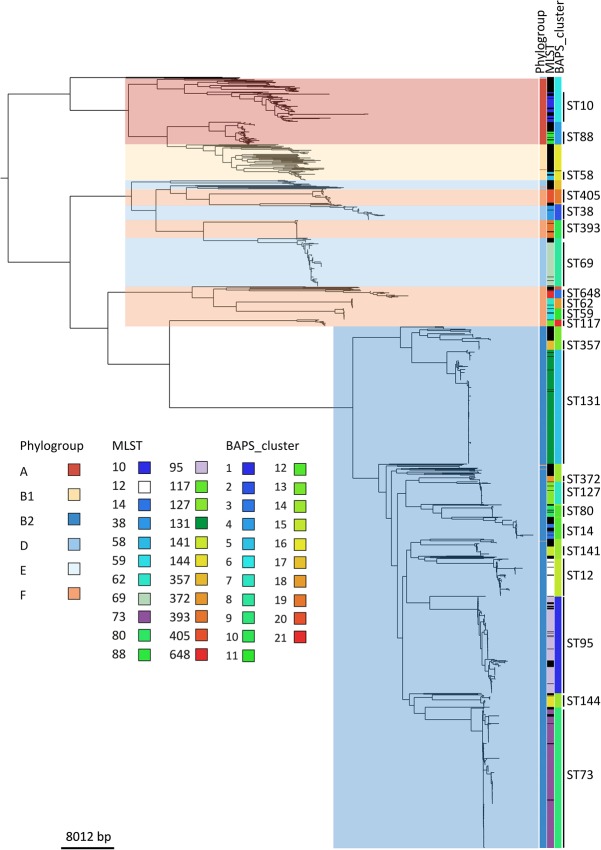
Maximum-likelihood core genome phylogeny of *E. coli* associated with bacteremia in England. The columns on the *right* show, from left to right, phylogroup, STs containing more than 10 isolates, and hierBAPS clusters. Phylogroups are also presented by background shading and STs labeled on the *right*. Black represents ST designation not shown due to these having fewer than 10 isolates. The root has been placed according to previous understanding of *Enterobacteriaceae* phylogeny.

The 1509 *E. coli* isolates were resolved into 228 STs. The most frequent STs were ST73 (17.3%), ST131 (14.4%), ST95 (10.6%), ST69 (5.5%), and ST12 (4.6%), which accounted for more than half of the collection. The distribution of STs between the BSAC and CUH collections was comparable. Details of all STs are provided in Supplemental Table S1 (Supplemental Figures and Tables S1). The population structure of the collection based on core genome single-nucleotide polymorphisms (SNPs) was defined using Bayesian analysis of population structure (BAPS), which provides an independent method of assessing the population structure based on the data in the collection, not based on previous definitions. This correlated well with ST (Fig. 1), and we therefore used STs to allow for direct comparisons between our data and previous studies. However, there were inconsistencies with phylogroups, which have been linked to the source of isolation and virulence (Picard et al. 1999) and have been previously used to describe the *E. coli* population structure (Lecointre et al. 1998). Most isolates (*n* = 1018, 67%) were assigned to phylogroup B2. The remainder were distributed among phylogroups F (*n* = 151, 10%), A (*n* = 130, 9%), D (139, 9%), B1 (*n* = 69, 5%), and E (*n* = 2, <1%) (Fig. 1). Four of the five most common STs resided in phylogroup B2 (ST73, ST131, ST95, and ST12), while ST69 belonged to phylogroup D. A comparison of ST, BAPS clusters, phylogroup, and a maximum likelihood (ML) tree based on core genome SNPs is shown in Figure 1. The phylogeny showed five large clades, which generally correspond to phylogroups. However, comparison between phylogroup and core genome-based phylogeny showed that phylogroups F and D were mixed rather than monophyletic groups (Fig. 1). This is consistent with the PCR data from Clermont et al. (2013), as well as the presence of an A genotype within the B1 group (Clermont et al. 2013). The ML tree was dominated by phylogroup B2, which showed large clonal expansions. These were mostly absent from groups A and B1, which were in turn dominated by isolates on long branches.

Analysis of the pan-genome demonstrated an open pan-genome containing 69,274 genes and no sign of reaching a plateau (Supplemental Fig. S1). By use of a strict definition of core genome, only 885 genes were present in all 1509 isolates, although this rose to 1744 genes using a cut-off of presence in 99% of isolates. The vast majority of genes (62,753 of 69,274, 91%) were present in <15% of the isolates.

### The population structure of *E. coli* associated with bacteremia

**Figure 2.**
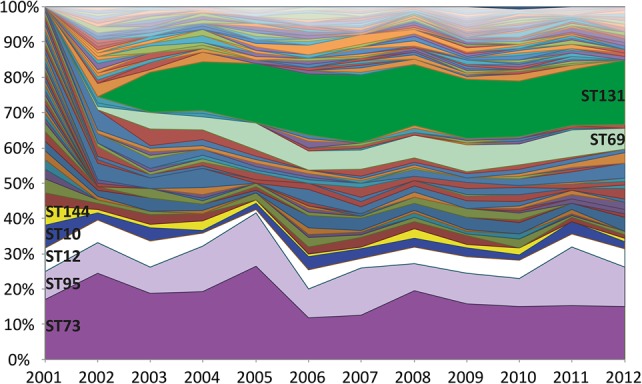
Proportions of STs during the 11-yr sampling framework. The percentage of each ST has been plotted by year ordered by the frequency at the start of the study (most common at the bottom). The emergence of ST131 and ST69 can be observed in 2003 and 2002, respectively.

Two STs appeared in the collection for the first time during the timeframe of the study, with ST69 first detected in 2002 and ST131 in 2003. The proportion of STs in each year of the collection is shown in Figure 2. Within a short period after the emergence and spread of ST69 and ST131, the population established a new equilibrium, whereby the proportion of the major STs remain relatively unchanged. The proportion of ST73, ST95, and ST12 before and after the emergence of ST131 was on average 24% versus 17%, 8% versus 11%, and 7% versus 4%, respectively. The proportion of the remaining STs fell from 59% before the emergence of ST131 to 42% after but was stable from then until the end of the sampling period.

### Genetic characterization of ST131

**Figure 3.**
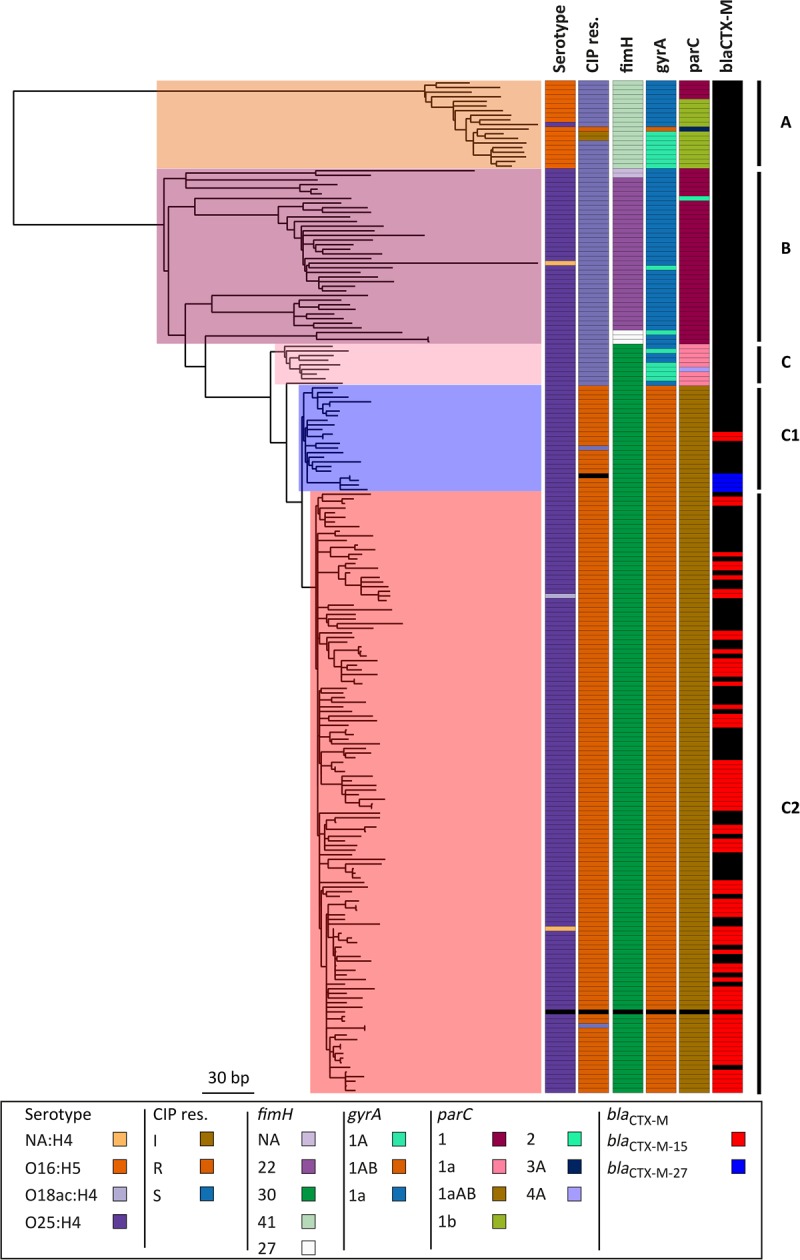
ST131 maximum-likelihood phylogenetic tree based on SNPs called against the reference EC958. Columns to the *right* of the tree show the in silico predicted serotype (O16-H5 or O25-H4); phenotypic resistance to ciprofloxacin (CIP res.); SNP-based definition of *fimH*, *gyrA*, and *parC* genotypes; and the presence of *bla*_CTX-M_ and the type. NA:H4 in the serotype indicates that we were unable to assign a definite O type for the isolate. It has not been counted as a new serotype. Clades assigned based on the markers and clade-specific SNPs are shown on the *right*. The only isolate with missing data (black) is the reference strain EC958. The tree is mid-point rooted.

Three major clades have been identified for ST131 (Price et al. 2013; Petty et al. 2014): Clade A corresponds to serotype O16:H5, clade B is serotype O25:H4 and is negative for the *fimH*30 allele, and clade C (H30) is serotype O25:H4 and is positive for *fimH*30 and subdivided by the acquisition of fluoroquinolone resistance in clade C1 (H30-R in Price et al. 2013). This has been further divided into clade C2 (H30-Rx in Price et al. 2013), described previously as having acquired *bla*_CTX-M-15_ encoding extended spectrum beta-lactamase (*ESBL*), followed by expansion of this clade (Price et al. 2013; Olesen et al. 2014). Our 218 ST131 isolates were assigned to these lineages using previously described lineage-defining variation. This demonstrated that 197 (90%) were serotype O25:H4, and 18 (9%) isolates at the base of the lineage were serotype O16:H5 and *fimH41*. For two isolates, the serotype could not be explicitly defined in silico, and one was defined as O18ac:H4. One O25:H4 isolate was within the O16:H5 positive clade A. The C1 clade was defined based on a comparative phylogenetic analysis with the Price et al. (2013) isolates (Supplemental Fig. S2) and in silico PCR to detect H30-Rx (C2)–specific SNPs. Of the 161 ST131 isolates in lineage C, 129 belonged to the C2 clade (Fig. 3). The assignment of isolates to clades was confirmed by investigating six previously reported clade specific SNPs for B, C, C1, and C2 (Ben Zakour et al. 2016). This confirmed our assignment of isolates to clades and revealed that the three *fimH*27 isolates in the B clade most likely belong to the B0 clade defined by Ben Zakour et al. (2016).

We then mapped *bla*_CTX-M-15_ and fluoroquinolone resistance across the ST131 collection. *bla*_CTX-M-15_ was present in both C1 and C2 clades but was not detected in clades A or B (Fig. 3). A parsimony reconstruction of the presence of *bla*_CTX-M-15_ within a EC958 reference genome-based ML phylogeny using both acctran and deltran methods indicated at least 28 introductions and/or losses of the *bla*_CTX-M-15_ gene in this data set. This indicates that *bla*_CTX-M-15_ has been acquired and lost repeatedly in the C1 and C2 clades (Supplemental Fig. S3). The majority of the *fimH*30-positive C isolates were ciprofloxacin (fluoroquinolone) resistant, with a small number of exceptions. A cluster of eight fluoroquinolone-susceptible isolates resided close to the root of the C clade, together with two sporadic isolates in the C1/2 clade. Altogether, 75 of ST131 isolates were *bla*_CTX-M-15_ positive. The C1 clade contained only 23 isolates, but of these, two were *bla*_CTX-M-15_ positive and another four had acquired *bla*_CTX-M-27_. Of the 129 clade C2 isolates, 73 were *bla*_CTX-M-15_ positive. Isolates in lineage A and B were mostly susceptible to ciprofloxacin, the exceptions being one isolate in clade A that was resistant to ciprofloxacin and two clade A isolates with intermediate resistance. The resistant isolate had the *gyrA* mutation associated with fluoroquinolone resistance (*gyrA*1AB) (Johnson et al. 2013).

### ST131 *espC* island

We analyzed the presence of 3511 virulence genes in the whole collection and observed that just one gene was almost specific to the ST131. This was present in ST131 and the closest lineages in the B2 phylogroup. This was more common in ST131 (*N* = 216, 99.08%) compared with other STs (*P*-value <2.2 × 10^−16^). The gene was annotated as *espC* (a member of the serine protease autotransporters of *Enterobacteriaceae*, [SPATE] family). The gene is contained in a genetic island reported previously as ROD3 in ST131 strain EC958 (Totsika et al. 2011), but is not identical to the first description of an *espC* pathogenicity island that was originally reported in EPEC (Stein et al. 1996; Mellies et al. 2001; Schmidt and Hensel 2004). The sequence identity/similarity of the EPEC *espC* and EC958 *espC* was 68% for DNA and 69%/73% for protein. The ST131 *espC* island has genes coding for *fimD*, *focC* (*fimC*), *tsh*, *cfaD* (*regA*), and *espC*, along with two poorly characterized proteins, one with similarity to fimbrial adhesins and one to DNA binding proteins. The island is bordered by *yjdJ* genes. The locus where the island is inserted is conserved at the *yjdIJKO* gene region, between the *dcu*S and *lysU* genes in *E. coli* reference strain K-12 MG1655 (NC_000913.2). Analysis of mapping coverage to the reference EC958 showed that the island was present in all ST131 isolates (*N* = 218), closely related clades to ST131 in the B2 phylogeny (*N* = 45), and four isolates in phylogroup D and six in phylogroups B1/A. A region of the island is missing from 11% of the ST131 isolates, 19 of which belong to the clade A and were missing a common region. The same region was missing in the clades close to ST131 in the phylogeny (Supplemental Fig. S4). The *espC* allele present outside of the ST131 clades B and C was often different than the *espC* in the rest of ST131 (Supplemental Fig. S5).

### ST73 and ST131 have different strategies to achieve prevalence

**Figure 4.**
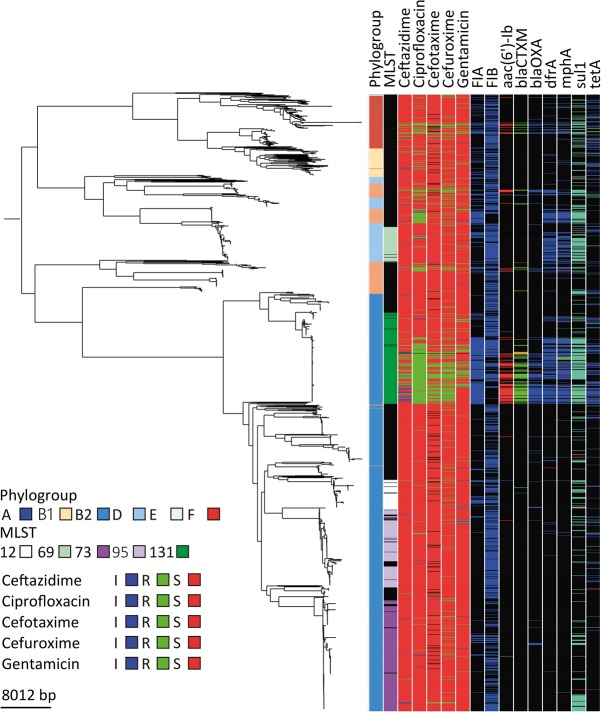
Multidrug-resistance plasmids present in ST131. Phylogeny of the whole collection with columns to the *right* representing phylogroup, the five most frequent STs, phenotypic antibiotic-resistance data linked to the plasmid (ceftazidime, ciprofloxacin, cefotaxime, cefuroxime- and gentamicin), the presence of incFIA and incFIB, and antibiotic-resistance genes carried by the plasmid (*aac(6′)-Ib*, *bla*_CTX-M_, *bla*_OXA_, *dfr*A, *mph*A, *sul*1, and *tet*A). (Black) Missing; (color) present. The phylogenetic tree is the same as in Figure 1.

ST73 and ST131 represented the predominant STs in the collection but are known to have contrasting antibiotic-resistance profiles. Consistent with this, our ST131 isolates were predominantly MDR, and ST73 was largely susceptible (Fig. 4; Supplemental Fig. S6). This was reflected by the presence of numerous antibiotic-resistance genes in ST131 compared with ST73, which was accounted for at least in part by different plasmid profiles. ST131 was the main lineage in the collection to carry an incFIA plasmid(s) (Fig. 4; Supplemental Fig. S6). This contained *aac6*′-lb-cr, *bla*_CTX-M-15_, and *bla*_OXA1_ and indicates that this plasmid is mostly responsible for the multidrug-resistant ST131 phenotype (Supplemental Fig. S6). A more widely disseminated plasmid in ST131 was also present and carried incFIB often in addition to incFIA. This plasmid encodes *bla*_TEM-1_, *dfrA*, *mphA*, *sul1*, and *tetA*. Due to the limitations of Illumina short-read technology, it is not possible to further delineate the structure of the genetic element encoding these genes, and therefore, the presence of genes in specific plasmids is determined by association alone and has a level of uncertainty. The difference in susceptibility profiles of ST73 and ST131, which remain at stable proportions of the population throughout the study after the introduction of ST131, suggests that resistance may not be the primary determinant of successful establishment and maintenance in the reservoir niche.

**Figure 5.**
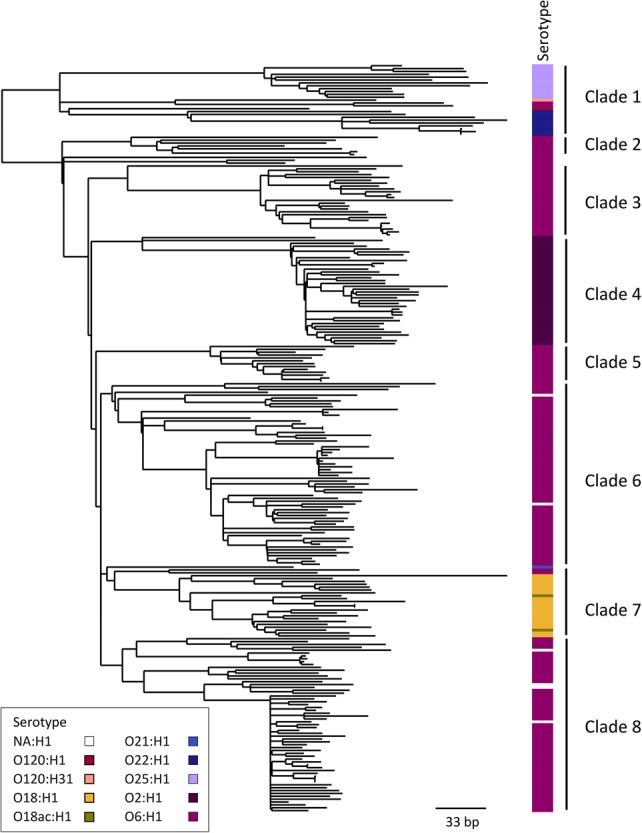
ST73 maximum-likelihood phylogenetic tree based on SNPs called against the reference CFT073. The in silico predicted serotype is shown to the *right* of the tree. For serotype NA:H1 the O type could not be assigned. This is not counted as a new serotype. The clades are labeled on the *right*. The tree is mid-point rooted.

Comparing the phylogeny of the two lineages showed that ST131 was mainly dominated by clade C isolates, which were very closely related, but the ST73 tree has several more divergent clades. The average pairwise SNP distance between all the ST131 isolates was 156 SNPs (median = 74 SNPs) (Fig. 3). In contrast, the ST73 phylogeny comprised at least eight clades with isolates that were much less closely related (average pairwise SNP distance in ST73 is 335 SNPs, median = 332 SNPs) (Fig. 5). This is underlined by the observation that ST73 isolates were assigned to nine serotypes in silico, and the different serotypes were in phylogenetically separate lineages. It seems likely that a change in serotype has occurred at least seven times in ST73 (Fig. 5). In contrast, within ST131 only three serotypes were identified. O16:H5 was present in clade A, and O25:H4 was present in the rest of the phylogeny represented by clades B and C. One isolate in the C2 clade was O18ac:H4. A comparison of the presence of virulence genes in ST73 and ST131 revealed differences in the presence of the UPEC/ExPEC virulence genes between the two (Supplemental Fig. S5), suggesting that, again, there is not a single configuration that is best for success in this niche represented by MDR ST131, but, rather, also susceptible, but fit and virulent, STs can become prevalent. For example, most ST131 isolates lacked gene clusters *hlyABCD* (hemolysin) and *iroBCDN* (salmochelin) but carried genes for aerobactin (*iucABCD*, *iutA*), hemin uptake (*chuASTUWXY*), and yersiniabactin (*fyuA*, *irp1, irp2*, *ybtAEPQSTUX*) (Supplemental Fig. S7). It seems that ST131 can use only aerobactin, yersiniobactin, and the *chuASTUWXY* for iron acquisition, but for example, ST73 has the potential to use all these and others.

An interesting similarity between the two most prevalent lineages ST131 and ST73 is that both are positive for *sat* (ST131 89% and ST73 87%), a gene encoding a secreted autotransporter toxin that is toxic for kidney and bladder cell lines (Guyer et al. 2000) and is less widely present in rest of the population (27%; *P*-values <2.2 × 10^−16^). Another similarity is the absence of *aec* genes in ST73 and ST131 (except *aec7*, which is present in ST73), which encode genes associated with type 6 secretion, although they were widely present in the rest of the phylogeny (Supplemental Fig. S5).

### Two strategies for emergence—ST131 and ST69

Two independent lineages (ST69 and ST131) became established in our study population during the study period. To investigate the structure and history of this emergence, we constructed temporally resolved phylogenies using Bayesian evolutionary analysis by sampling trees (BEAST). Based on the BEAST analysis, the MRCA (most recent common ancestor) for ST69 was dated to 1956 (95% highest posterior density [HPD] interval, 1935–1971). This separated the major clade in the ST69 tree that subsequently divided into two large lineages in 1977 (95% HPD interval, 1965–1986) (Supplemental Fig. S8). By using the Bayesian skyline model, we could estimate the effective population size in the past. The analysis showed three increases in the population size. The first increase beginning in the late 1970s and the second in the 1990s were smaller than the last rapid increase that occurred relatively close to the year 2000 (Supplemental Fig. S9). If the confidence interval is taken into account, we hypothesize that we observed the last increase in population size during this study.

**Figure 6.**
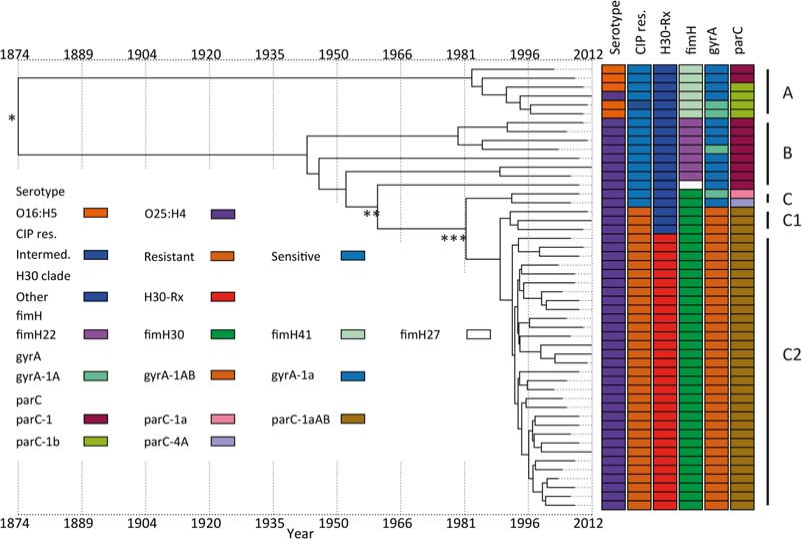
Temporal analysis on ST131 using BEAST. Figure shows the serotype, resistance to ciprofloxacin (CIP res.), assignment to clade C2 (H30-Rx), and the gene alleles of *fimH*, *gyrA*, and *parC*. (*) MRCA; (**) emergence of clade C; (***) emergence of CIP-resistant clade C1.

The BEAST analysis estimated that the MRCA of the clades with the two different serotypes in ST131 was around 1874 (95% HPD interval, 1697–1951) (Fig. 6; Supplemental Fig. S10). The C clade diverged from the rest of the phylogeny around 1960 (95% HPD interval, 1899–1985), and the fluoroquinolone-resistant C1 clade is estimated to have diverged from the rest of the ST131 lineages around 1982 (95% HPD interval, 1948–1995). We repeated this analysis with least-squares dating (LSD), which gave a date for the MRCA of the complete ST131 collection of 1828 (confidence interval 1672–1891), 1934 (1871–1960) for divergence of clade C from A and B clades, 1979 (1953–1991) for divergence of the fluoroquinolone-resistant clade from the rest, and 1986 (1965–1993) for the divergence of C2 clade from C1. The majority of the ST131 nodes, including the fluoroquinolone-susceptible lineages in the tree had a divergence time of ≤30 yr. This is indicative of the fact that the whole ST131 lineage, rather than just a single clade within it, has increased in prevalence after its observed emergence in England in the 2000s. This is also apparent from an analysis of isolation dates against the phylogeny of ST131 in our collection (Supplemental Fig. S11). It can be seen that nearly all of the major clades of the tree contained isolates from every year, indicating that the whole population was present in each year throughout the 11 yr in this study, not just the clade C2. The skyline plot showed a sharp increase in the population size around the year 2000 (Supplemental Fig. S9). This most likely correlates with the emergence of the ST131 lineage that we observed in our data set.

### Distribution of virulence factors

We analyzed the repertoire of virulence factors, focusing on genes present or absent in the most prevalent sequence types in our collection, ST73 and ST131. ST131 is known to cause UTIs, yet this clade has only a partial *pap* (P fimbriae or pyelonephritis associated pilus) gene operon (Clark et al. 2012): 82% of ST131 had only *papABIX* or fewer genes from the operon and not, for example, the tip adhesin *papG*. In more detail, 90% of ST131 isolates had *papA*, but only 17% had the tip adhesin *papG* (Supplemental Fig. S7). Most of the other clades in the B2 phylogroup contained the intact operon, which was also present in other phylogroups. In addition, the most closely related clades to ST131 were also missing most of this operon. Genes encoding enterotoxins *set1AB*, autotransporter *pic*, F1C fimbrial genes *focAGH*, and the autotransporter *upaH* (Supplemental Fig. S5) were found in ST73 but were rare in other STs.

Mapping against the reference strain EC958 also enabled us to analyze the presence of the reported ST131 genomic island ROD3 (Totsika et al. 2011) and the type 6 secretion system (T6SS) across the whole invasive *E. coli* population in England (Supplemental Fig. S4). T6SS was specific to the B2 phylogroup. The T6SS is used as an anti-competition mechanism to enhance survival in a competitive niche such as the gut (Chatzidaki-Livanis et al. 2016; Sana et al. 2016). This may be one explanation for the prevalence of the B2 phylogroup in our collection.

### Antibiotic resistance

**Figure 7.**
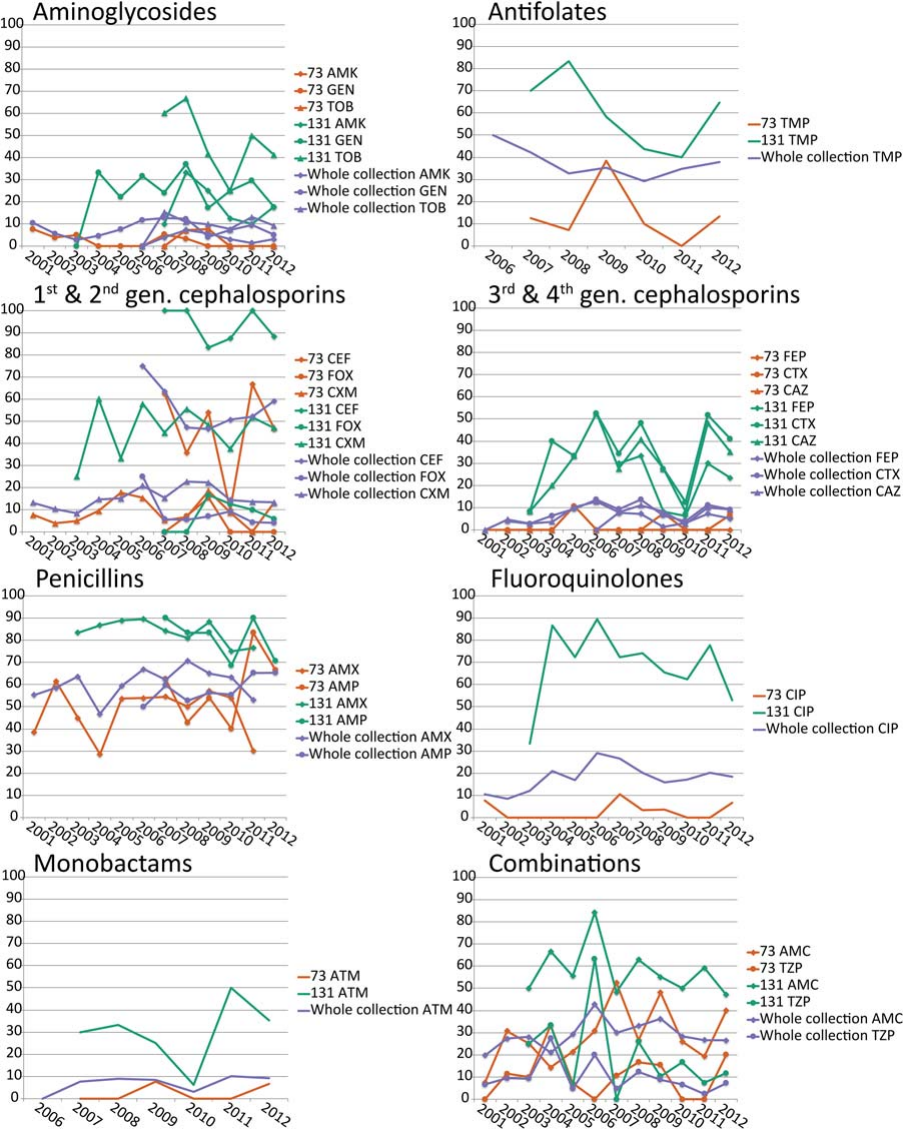
Comparison of antibiotic resistance of ST131, ST73, and the whole collection. Phenotypic antibiotic-resistance data are represented by the percentage of nonsusceptible (resistant + intermediate) isolates per year. Each subfigure represents one antibiotic class. Carbapenems (imipemen, meropenem, and ertapenem) and tigecycline are not shown due to the lack of resistance against these classes in this collection. (AMK) Amikacin; (GEN) gentamicin; (TOB) tobramycin; (TMP) trimethoprim; (CEF) cefalotin; (FOX) cefoxitin; (CXM) cefuroxime; (FEP) cefepime; (CTX) cefotaxime; (CAZ) ceftazidime; (AMX) amoxicillin; (AMP) ampicillin; (CIP) ciprofloxacin; (ATM) aztreonam; (AMC) amoxicillin-clavulanic acid; (TZP) piperacillin-tazobactam.

Phenotypic antibiotic resistance to ciprofloxacin increased from 10.5% to 28.8% (probably due to the emergence of ST131) and peaked in 2006, but it remained under 20% from 2008–2012. It is striking that there was no consistent change in the phenotypic antibiotic resistance of the two most prevalent sequence types, ST73 and ST131, except for ampicillin resistance in ST73 (Fig. 7). Furthermore, there was no clear increase in antibiotic resistance over time for the whole collection. ST131 was more resistant to most antimicrobials than ST73 (and often the rest of the collection) and contained the most antibiotic-resistance genes (Fig. 4). However, the equally successful ST73 was one of the least resistant lineages to most antimicrobials. Phenotypic antibiotic susceptibility results are summarized in Table 1.

**Table 1.**
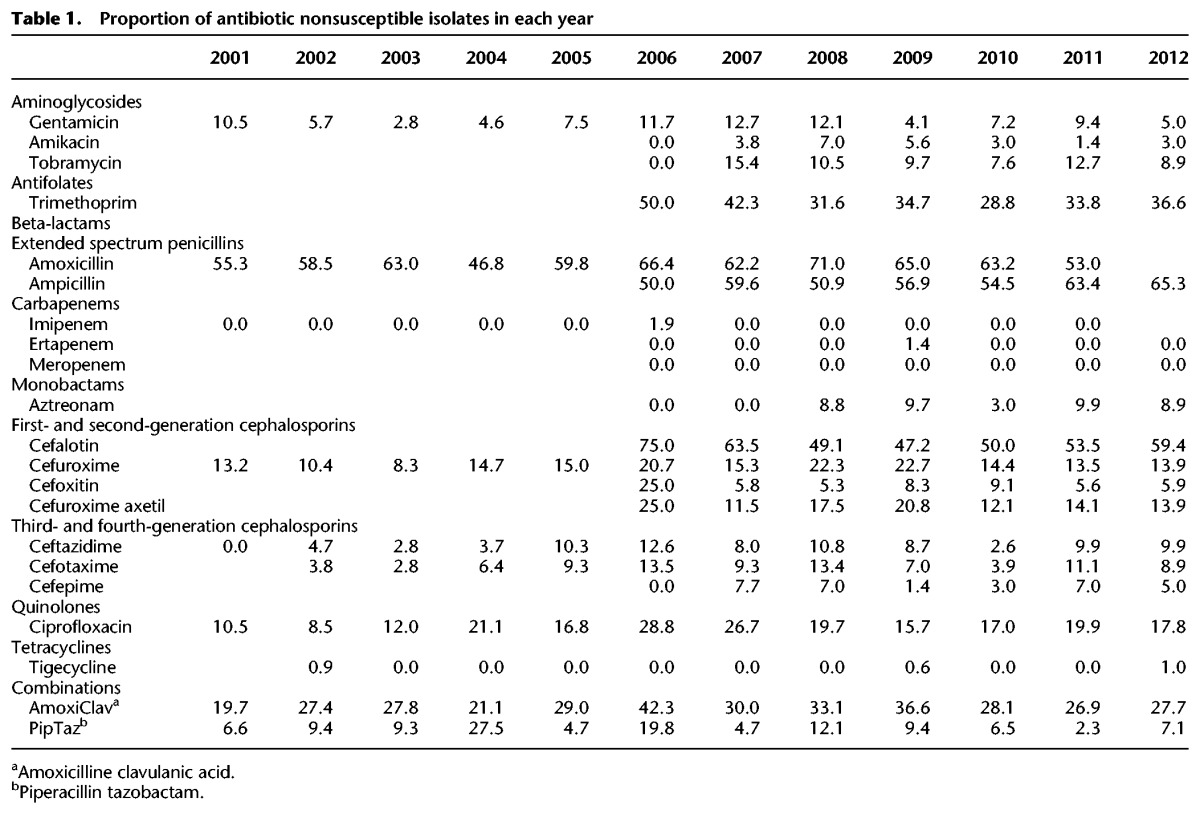
Proportion of antibiotic nonsusceptible isolates in each year

## Discussion

We analyzed WGS data for 1509 *E. coli* blood isolates taken from a systematic sentinel-based surveillance program across England, as well as unbiased sampling from a university hospital, isolated in 2001–2012. This 11-yr period enabled us to analyze the temporal trends in the population structure and changes in the antibiotic resistance of invasive *E. coli*, as well as characterize in detail the most prevalent sequence types causing bacteremia. The most predominant STs were ST73, ST131, ST95, ST69, and ST12. These belong to *E. coli* phylogroups B2 and D, which have been previously associated with virulent and pathogenic UPEC and ExPEC strains (Picard et al. 1999; Johnson and Stell 2000; Johnson et al. 2001). The fact that some of the genotypes from the (in silico) PCR did not present monophyletic clades made it difficult to assign all isolates to phylogroups without the use of a phylogenetic tree. The pattern of prevalence of STs is consistent with previous studies of isolates associated with urinary sepsis or bloodstream infection; for example, during a similar time period in Ireland the most frequent STs were ST131, ST73 and ST69 (Miajlovic et al. 2016) and the *E. coli* from the BSAC Bacteraemia Resistance Surveillance Programme have the same most common profiles (CC73, CC131 and CC95) as this combined collection from BSAC and CUH (Day et al. 2016). This also shows that the collections from BSAC and CUH are similar when STs are considered. Information from hierBAPS clustering showed that BAPS clusters were more often monophyletic than STs since single and double locus variants often disturbed the uniformity of ST clades. The presence of phylogroup F isolates within group D, already reported in the original article presenting the method (Clermont et al. 2013), made the use of the current PCR-based method for assigning isolates to phylogroups problematic for some clades.

The study period captured the emergence of ST69 and ST131 into our study population, but the introduction of these lineages only disturbed the population structure transiently, after which it quickly reached a new equilibrium, with the new lineages subsequently maintaining a stable proportion of the population. Despite its apparent success, the globally disseminated MDR lineage ST131 failed to become dominant in the whole population. The most common lineage prior to the emergence of ST131, ST73, only reached a proportion of >20% of the whole population in a single year. This suggests that the driver for the overall structure of the population and proportions of successful clones may be a form of negative frequency-dependent selection. New STs have an advantage when rare, but this is lost when they become more common. One hypothesis to explain this is that the *E. coli* causing bloodstream infections do not form a discrete population but represent a spill-over of *E. coli* that occupies a commensal niche in the wider human population. This is supported by evidence that both drug-resistant (ST131) and drug-susceptible (ST73) lineages are equally successful in being maintained in this reservoir and that drug resistance as a whole in the population is not increasing, demonstrating that antibiotic resistance is not a primary driver of success or prevalence in this niche. In the case of ST131, this is supported by the findings of Ben Zakour et al. (2016), who reported that virulence determinants were acquired before the emergence of the fluoroquinolone-resistant C clade (Ben Zakour et al. 2016). According to this hypothesis, the primary forces shaping the population are not those within the hospital environment but are due to competition within the gut commensal niche in the broader human population, where antibiotic use is more sporadic than in the hospital population. This is a strong contrast with the population structure of true nosocomial pathogens such as methicillin-resistant *Staphylococcus aureus* (MRSA), where specific drug-resistant clones sequentially dominate the population within a niche where antibiotic exposure is common and drug resistance is a strong selective advantage.

ST73 in our collection was susceptible to the antibiotics tested, but a recent report from the United Kingdom found several MDR ST73 isolates associated with an MDR plasmid (Alhashash et al. 2016). Further studies are needed to define whether this MDR phenotype will become more disseminated in ST73 over time.

Although previously reported to be present in the United Kingdom and Ireland from 2001 (Day et al. 2016), we observed the appearance of ST69 and ST131 within our sampling framework. We used BEAST to analyze their emergence and found that the events leading to their spread seem quite different. A Bayesian skyline analysis of ST69 showed several sequential minor increases in population size, which started in the late 1970s with the last and most rapid one coinciding with the beginning of this study. The most recent common ancestor of ST69 could be dated to ∼60 yr ago. A similar analysis of the ST131 lineage identified that the split between the O25:H4 and O16:H5 lineages occurred ∼143 yr ago. The phylogeny and the Bayesian skyline analysis support the observation that there was a single rapid expansion of this clade in the last few years, starting around 1995. However, rather than being due to a single sublineage, this expansion seems to have happened in the entire O25:H4 clade and possibly the whole ST. According to our analysis, the *fimH30* carrying clade C diverged from clade B around 1960, the fluoroquinolone-resistant clade C1 (H30-R) diverged from clade C around 1982, and the clade C2 (H30-Rx) diverged from the rest of clade C around 1990. These dates are somewhat different from the recently published analyses of Stoesser et al. (2016) and Ben Zakour et al. (2016). The emergence of the fluoroquinolone-resistant subset of the C clade in the 1980s found here is consistent with two previous publications. Similarly, the divergence of the C2 clade from the C1 in 1990 reported here is similar to the year of 1987 reported by Ben Zakour et al. (2016) and Stoesser et al. (2016). This indicates a strong temporal signal in the sequences for these genetic events. The difference in other dates are most likely due to weaker temporal signals in the data for these events, for example, the divergence of the C clade from B clade is dated close to the year 1960 by Ben Zakour et al. (2016) and in this study but to 1985 by Stoesser et al. (2016). The fact that all of the ST131 clades were present during the whole study period indicates that the entire ST131 lineage is successful and not just clade C or the fluoroquinolone-resistant and *ESBL*-expressing clades C1 and C2.

For ST131, the presence of *bla*_CTX-M-15_ was initially reported to be exclusive to the H30-Rx (C2) clade (Price et al. 2013). In our collection, *bla*_CTX-M-15_ was found in both the C1 and C2 clades. We also identified that *bla*_CTX-M-15_ was acquired and/or lost several times in the ST131 population studied here. Stoesser et al. (2016) had previously hypothesized that this could occur based on the diverse contexts in which the gene is found. It is also noteworthy that our unbiased sample of *E. coli* causing BSI was dominated by C2 isolates, with >80% of clade C isolates and ∼60% of all the isolates belonging to clade C2. This is in contrast to the previous reports of the ST131 population structure where the C1 and C2 have been more equally distributed (Price et al. 2013; Petty et al. 2014; Ben Zakour et al. 2016; Stoesser et al. 2016).

The pan-genome of our collection of invasive *E. coli* included almost 70,000 genes. The core was very small, and most of the genes in the pan-genome were present in a small subset of strains, reflecting the massive diversity of *E. coli*. Previous reports on the size of the *E. coli* pan-genome have been markedly smaller, but also, the data sets that the analyses were performed on have been smaller. With an open pan-genome, this will have an effect on the pan-genome size. Rasko et al. (2008) reported a pan-genome of over 13,000 genes and a core of 2200 genes from an analysis of a diverse set of 17 genomes from different *E. coli* pathovars. Chen et al. (2006) analyzed the pan-genome and core genome of seven UPEC isolates and reported a core genome of 2865 genes. Both of these estimates of the core are considerably larger than ours. This may be due to the high similarity cut-off in our analysis, which will make the core genome appear smaller and the pan-genome larger. However, even with a cut-off of 90% the pan-genome is 46,022 genes in this data set and the core genome 1170 genes. Recently, a core of 1080 gene clusters was reported in a study investigating 70 EPEC isolates using large-scale BLAST score ratio analysis (Hazen et al. 2016). This is closer to our number of 885 core genes even though the pan-genome in the analysis by Hazen et al. (2016) was only 12,964 gene clusters. This is likely due to the different methods that were used in the analyses.

The proportion of antibiotic-resistant isolates in the data set did not increase substantially during the 11 yr that were included in this study. The resistance patterns fluctuated over time, which was probably due to the relatively small sample size per location per each year. Despite the fact that we did not observe a clear increase in the proportion of resistance, there have been many recent reports suggesting an increase in the proportion of resistant isolates (ESPAUR 2015; Ironmonger et al. 2015). This may be due to differences at the regional level, while this study was performed at a nationwide level, and therefore, smaller increases at a local level might not have been apparent.

The analysis of virulence factors in this diverse population enabled us to compare the most successful lineages to each other and to the rest of the population. We identified a number of virulence factors that were differentially present between lineages. One gene, the SPATE *espC*, was specific to ST131 and closely related lineages. This is situated in a pathogenicity island that is restricted almost exclusively to ST131 and its closest clades. This island was first reported by Totsika et al. (2011) as a region of difference 3 (ROD3), and has previously been reported to be present throughout ST131 by Petty et al. (2014) but not to be conserved in clade A (Totsika et al. 2011; Petty et al. 2014). Since the ST131 *espC* island does not have an integrase or other mobility-related genes, it is not clear if it is a self-mobile element. Its sporadic acquisition in different branches of the tree could reflect acquisition by mechanisms other than self-mobility (such as homologous recombination in flanking sequences) or could potentially represent lineage-specific deletion. In EPECs, *espC* has been shown to play a role in cell death by causing apoptosis and necrosis (Serapio-Palacios and Navarro-Garcia 2016), and the ST131 *espC* island has recently been reported to harbor a gene encoding the regulatory protein RegA (annotated as *cfaD* by Prokka in our analysis) that is present in *Citrobacter rodentium* and *Escherichia* clades III, IV, and V (Tan et al. 2015). Incorporation of this island may be one reason for the success of ST131. There were several genes present almost exclusively in ST73 that could in part contribute to its success. These genes were *focAGH* (encoding F1C fimbriae genes), *pic* (encoding another SPATE gene), *set1AB* (encoding *Shigella* enterotoxin 1), and *upaH*, which is an autotransporter that induces biofilm formation and bladder colonization (Allsopp et al. 2010, 2012). The secreted autotransporter toxin encoded by *sat* (another SPATE gene) was present in both ST131 and ST73 and could be contributing to the success of both lineages.

One limitation of this study is that although the collection was drawn from 11 centers over 11 yr, it only comprised the first 10 isolates per site each year except in the case of CUH. It could therefore potentially include isolates from temporally limited local epidemics, which could skew the results and interpretation. In addition, the limitation of short-read sequencing is evident when analyzing plasmids. The dynamic nature of plasmids, in combination with short reads generated by the Illumina HiSeq, means that many differences in plasmid structure are unlikely to be captured by our analyses. We are also unable to assemble complete plasmid sequences, and so the presence of genes in plasmids with given inc-types is based on association alone. More detailed analysis would require the use of long-read technologies. Determining the presence of genes by association alone adds a degree of uncertainty to the results due to untypeable plasmids and the mobile nature of the genetic elements that can be present in the chromosome.

In summary, we have analyzed the population structure of *E. coli* associated with bloodstream infection over an 11-yr period in England. During this time, we observed the emergence of ST131 and ST69, but this introduction did not disturb the population structure for long, and a new equilibrium was quickly established. The globally disseminated MDR lineage ST131 was not the most frequently identified lineage in this collection; this was ST73, which is generally susceptible to most antibiotics. This indicates that antibiotic resistance is not a primary driver of success in the niche occupied by these *E. coli*. The relatively static structure of the population suggests that it is instead driven by negative frequency-dependent selection occurring in the commensal niche in the wider human population and that bacteremia represents a spill-over from this population. This emphasizes the importance of surveillance of the wider human population to understand the dynamics and structure of invasive *E. coli*.

## Methods

### Bacterial isolates

A total of 1522 *E. coli* isolates were initially included in the study. Of these, 1098 were from the BSAC Bacteraemia Resistance Surveillance Programme (www.bsacsurv.org) (Reynolds et al. 2008) between 2001 and 2011 (Supplemental Table S2). Up to 10 isolates (when available) were obtained for each year from 11 contributing laboratories distributed across England. The 11 centers were selected in order to provide geographical and temporal diversity. A further 424 isolates were sourced from the diagnostic laboratory at the CUH. Using the laboratory database, we selected every third isolate associated with bacteremia that had been stored in the −80°C freezer archive between 2006 and 2012. Thirteen isolates were subsequently excluded (four CUH isolates and nine BSAC isolates) based on the low quality of sequence data or species misidentification, giving a final sample size of 1509 isolates. Antimicrobial susceptibility testing was performed using the Vitek2 instrument with the N206 card (bioMerieux) for isolates from the CUH and using the agar dilution method for the BSAC collection (Andrews 2001). For the purposes of this analysis, we combined phenotypic antibiotic-resistance data from BSAC and CUH and grouped together the intermediate and resistant isolates in the analyses to represent the nonsusceptible part of the population. Since the isolates from the BSAC and CUH have been tested against different antibiotic combinations, we have antibiotic resistance data from 2001–2011 for amoxicillin and imipenem; from 2006–2012 for amikacin, tobramycin, ampicillin, ertapenem, meropenem, aztreoman, cefalotin, cefoxitin, cefepime, and trimethoprim; and throughout the study period (2001–2012) for gentamicin, tigecycline, cefuroxime, ceftazidime, cefotaxime, ciprofloxacin, amoxicillin-clavulanic acid, and piperacillin-tazobactam.

The National Research Ethics Service (ref. 12/EE/0439) and the CUH Research and Development (R&D) Department approved the study protocol.

### DNA extraction and sequencing

Genomic DNA was extracted using a QIAxtractor (Qiagen), and library preparation was performed according to the Illumina protocol. Index-tagged libraries were created, and 96 isolates multiplexed per lane and sequenced using the Illumina HiSeq 2000 platform (Illumina) to generate 100-bp paired-end reads. The average sequencing depth was 77-fold, with a minimum of 48-fold.

### Sequence data analysis

MLST was performed using an in-house script (Page et al. 2016b; https://github.com/sanger-pathogens/mlst_check) and STs defined using the Warwick MLST scheme (Wirth et al. 2006). De novo assembly was performed using Velvet (Zerbino and Birney 2008), and scaffolds were generated using SSPACE (Boetzer et al. 2011) and GapFiller (Boetzer and Pirovano 2012). Reads were mapped back to the assemblies using SMALT 0.7.5 (http://www.sanger.ac.uk/science/tools/smalt-0). Assemblies were annotated with an in-house pipeline based on Prokka (Seemann 2014). Annotated assemblies were used in a pan-genome analysis in Roary, from which a core genome alignment was generated (Page et al. 2015). Lists of genes in the core genome and the core genome alignment are presented in Supplemental Data S2 and S3, respectively. A fasta file of representative gene sequences of genes in the pan-genome is shown in Supplemental Data S4. These files are also available from figshare (see “Data access”).

Bayesian analysis of population structure (hierBAPS) (Corander et al. 2008; Cheng et al. 2013) was used to analyze the population structure. A core genome alignment was produced with Roary, and a SNP alignment was generated using SNP sites (Page et al. 2016a) and used in hierBAPS, which was run five times with the prior upper bound for the number of clusters varying between 100 and 300. All runs converged to the same estimate of the global posterior mode partition, indicating a strong support for the obtained clustering solution. Phylogenetic trees were generated using SNP sites determined by SNP sites from the core genome alignments or from SNPs identified by mapping to reference genomes, using RAxML 7.8.6 with 100 bootstraps (Stamatakis 2006). For the reference-based SNP tree, the sequences were mapped against a selected reference genome using SMALT 0.7.5 (http://www.sanger.ac.uk/science/tools/smalt-0), and SNPs were called using SAMtools (Li et al. 2009). Reference genomes used in the analyses were CFT073 (AE014075.1) and EC958 (NZ_HG941718.1) (Forde et al. 2014) for ST73 and ST131, respectively. Phage sequences were recognized using PHAST (Zhou et al. 2011) and were masked from the analysis. PHAST results can be retrieved from the program website (phast.wishartlab.com). Gubbins with default settings was used to identify recombination (Croucher et al. 2015), and the regions detected were masked from the alignment. The resulting alignment was used to produce a phylogenetic tree with RAxML.

Temporal analysis of ST69 and ST131 was performed with BEAST 1.7.5.1-1 (Drummond and Rambaut 2007; Drummond et al. 2012) on a reference-based alignment of 50 randomly selected isolates from both STs. This approach was used since BEAST did not converge with the complete collections or 100 isolates in the available running time on the computer cluster. References in the analysis were UMN026 (NC_011751.1) and EC958 (NZ_HG941718.1) for ST69 and ST131, respectively. PHAST was used to identify phage regions and Gubbins was used to identify regions of recombination in the alignment, and these regions were masked from the alignment before running BEAST. The nucleotide substitution model used was GTRGAMMA, and we ran three replicates of all combinations for strict clock and lognormal relaxed clock and three tree priors, coalescent: constant population, exponential growth, and Bayesian skyline. To estimate what was the best fitting model for each ST, we compared Bayes factors from marginal likelihood estimations calculated by path and stepping-stone sampling (Baele et al. 2012, 2013). Only the models that converged well and had an effective sample size (ESS) over 200 for each parameter were included in the test. The best fitting model was used in the subsequent analyses. For ST131, this was the Bayesian skyline model under the log-normal relaxed clock, and for ST69, it was the constant population model under the strict clock followed by the Bayesian skyline model under the strict clock. For the construction of the Bayesian skyline for ST69, we used data generated with the Bayesian skyline model under the strict clock. The temporal analysis for ST131 was confirmed with LSD (version 0.3 beta) using all isolates (To et al. 2016).

In silico PCR was used to assign isolates to *E. coli* phylogroups A, B1, B2, D, E, and F using the Clermont method (Clermont et al. 2000, 2013) to assign ST131 isolates to the B, H30-R (C1), and H30-Rx (C2) clades (Price et al. 2013; Ben Zakour et al. 2016) and to perform plasmid incompatibility group/replicon typing (Carattoli et al. 2005). Primers designed to detect clade-specific SNPs reported by Ben Zakour et al. (2016) are presented in Supplemental Table S3. In silico serotyping was performed with SRST2 according to the investigator's instructions with the database provided (Ingle et al. 2015). Here, serotype is defined by presence of known genes encoding serotype-determining enzymes. The results required minor manual curating when the gene typing resulted in a discrepancy between the gene pairs defining the serotype. This occurred mostly with the novel sequences the investigators had included in the database based on their own results. Antibiotic-resistance genes were detected using SRST2 with 98% identity. An in-house curated database based on ResFinder of antibiotic-resistance genes was used as reference (Zankari et al. 2012). Parsimony reconstruction of the presence of *bla*_CTX-M-15_ in ST131 was performed with the Fitch algorithm (Fitch 1971). Virulence genes were analyzed with SRST2 using the database and protocols described by the investigators and using an *Escherichia*-genus-specific database clustering together genes with 90% similarity and detecting genes with 90% identity and at least 90% coverage (Inouye et al. 2014). Gene typing for *gyrA*, *parC*, and *fimH* was performed by clustering sequences acquired from Roary using USEARCH (Edgar 2010). Clustering was based on reference sequences for seven *fimH* genes, seven *gyrA* genes, and 10 *parC* genes. The alleles tested were as described previously (Johnson et al. 2013) with the following exceptions: *fimH*15 was omitted and *fimH*31 was added to the analysis (Johnson et al. 2013).

### Statistical testing

To test if a specific gene is more often found in certain STs, we used Pearson's χ^2^ test statistic using prop.test in R (R Core Team 2015).

## Data access

Sequence reads from this study have been submitted to the European Nucleotide Archive (ENA; www.ebi.ac.uk/ena) under the accession numbers listed in Supplemental Data S1. The lists of genes present in the core genome and core genome alignment and representative sequences for the core genes are available in the Supplemental Data S2 through S4 and can be downloaded from figshare (https://figshare.com/s/20dfe5842f952497619b and https://figshare.com/s/3a12b011ff3c291a271b).

## Supplementary Material

Supplemental Material
