# Supplementary material for: Systematic longitudinal survey of invasive Escherichia coli in England demonstrates a stable population structure only transiently disturbed by the emergence of ST131
Source: Genome Res. 2017 Aug;27(8):1437–49. doi: 10.1101/gr.216606.116 (PMC5538559; doi:10.1101/gr.216606.116)
Supplement: Supplemental Material [file supp_27_8_1437__index.html]

Systematic longitudinal survey of invasive Escherichia coli in England demonstrates a stable population structure only transiently disturbed by the emergence of ST131 — Systematic longitudinal survey of invasive Escherichia coli in England demonstrates a stable population structure only transiently disturbed by the emergence of ST131 — Supplemental Material 

# Systematic longitudinal survey of invasive *Escherichia coli* in England demonstrates a stable population structure only transiently disturbed by the emergence of ST131

## Supplemental Material

- Supplemental\_Figures\_and\_Tables\_S1.pdf
- Supplemental\_Data\_S1.csv
- Supplemental\_Data\_S2.csv
- Supplemental\_Data\_S3.csv
- Supplemental\_Data\_S4.zip
