# Supplementary material for: Systematic longitudinal survey of invasive Escherichia coli in England demonstrates a stable population structure only transiently disturbed by the emergence of ST131
Source: Genome Res. 2017 Aug;27(8):1437–49. doi: 10.1101/gr.216606.116 (PMC5538559; doi:10.1101/gr.216606.116)
Supplement: Supplemental Material [file supp_gr.216606.116_Supplemental_Figures_and_Tables_S1.pdf]

Systematic longitudinal survey of invasive *Escherichia coli* in England demonstrates a stable population structure only transiently disturbed by the emergence of ST131

Teemu Kallonen, Hayley J. Brodrick, Simon R. Harris, Jukka Corander, Nicholas M. Brown, Veronique Martin, Sharon J. Peacock, Julian Parkhill

Supplemental tables and figures.

Contents Page

Supplemental Table S1 1
Supplemental Table S2 2
Supplemental Table S3 2
Supplemental Figure S1 3
Supplemental Figure S2 4
Supplemental Figure S3 5
Supplemental Figure S4 6
Supplemental Figure S5 7
Supplemental Figure S6 8
Supplemental Figure S7 9
Supplemental Figure S8 10
Supplemental Figure S9 11
Supplemental Figure S10 12
Supplemental Figure S11 13
References 14

Supplemental Table S1. Comparison of the collections based on MLST

ST	Combined collections	BSAC	CUH
**73**	17.3	17.8	15.9
**131**	14.4	13.3	17.3
**95**	10.6	10.3	11.3
**69**	5.5	4.8	7.5
**12**	4.6	5.1	3.1
**Top 5**	52.4	51.4	55.2
**Other**	47.6	48.6	44.8

Supplemental Table S2. Collections

	Number of isolates	Years of isolation
**CUH**	415*	2006–2012
**BSAC**	1094**	2001–2011
**Total**	1509	

*Number before QC was 434

**Number before QC was 1098

Supplemental Table S3. Primers to detect clades specific SNPs reported by Ben Zakour et al.

Target gene/CDS	fwd/rev	Primer sequence 5' to 3'*	Product length bp	Target position in product	Target position on reference genome EC958**	Clade specificity	Nucleotide change
ftsX	Fwd	TATCCAGCGCACCACCAAAA	494	312	3892567	B0, C0, C	A->G
	Rev	GGCCATGAATAAGCGCGATG					
EC958_0846	Fwd	AACACAACTGTTCGACTGCG	420	224	779389	B0, C0, C	C->T
	Rev	CCACTCTTCTTTTGCTGGCG					
kefC	Fwd	CTTTGCAGAGATTGGCGTGG	436	271	53758	C0, C	T->A
	Rev	GACATAGCGCCCCAGCAATA					
yqjA	Fwd	CGCATTATCATCAACGCGCA	453	229	3549826	C0, C	A->T
	Rev	TAGACTCTTGCTGGCGAACC					
iscS	Fwd	CATCTCGGTCGCCATCTCTT	484	256	2838072	C2	G->A
	Rev	GGTACTGGATACCTGCCGTC					
rmuC	Fwd	TATTCCCGTTGAACCCGCTT	488	243	4397873	C1	G->A
	Rev	GCTAGCGCGACTGCTGATTA					

*Designed using Primer-Blast (Ye et al. 2012)
** (Ben Zakour et al. 2016)

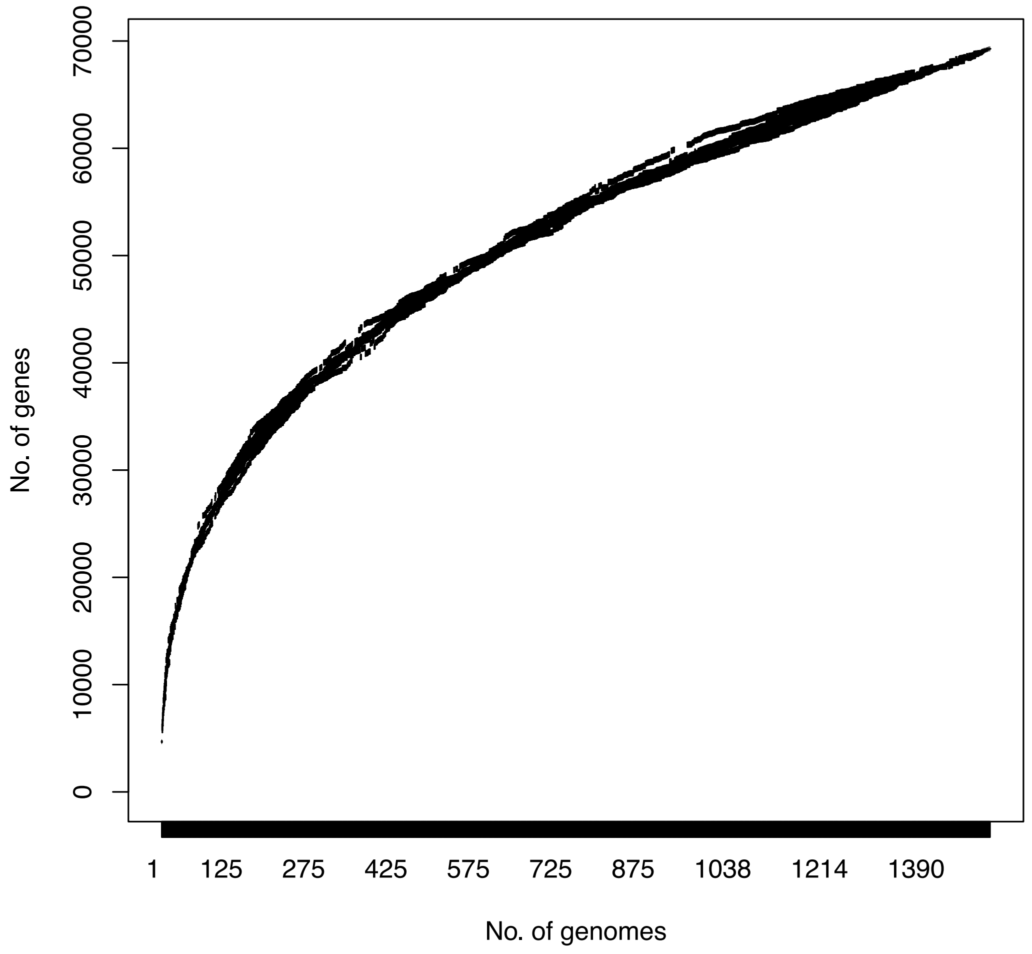

Supplemental Figure S1. Pan-genome of invasive *E. coli*. The y-axis presents the number of genes in the pan-genome and the x-axis addition of new genomes to the analysis.

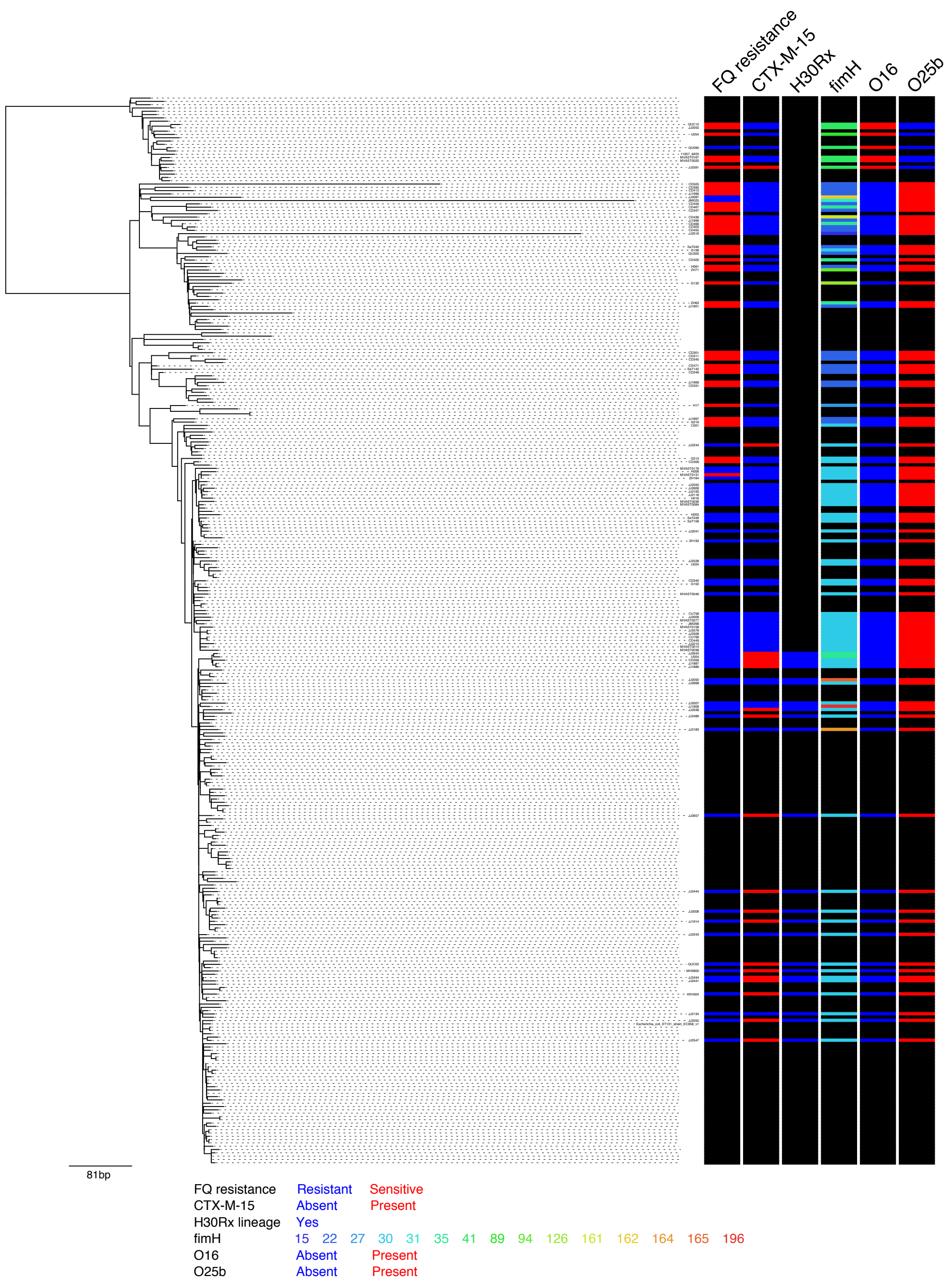

Supplemental Figure S2. Phylogenetic comparison of the data from this study with data from Price et al. 2014. (Price et al. 2013) In the figure are shown (from left to right) the fluoroquinolone resistance, presence of bla$_{CTX-M-15}$, inclusion to the clade C2 (H30-Rx), *fimH* type, serotype O16 or O25b. Black represents data not shown.

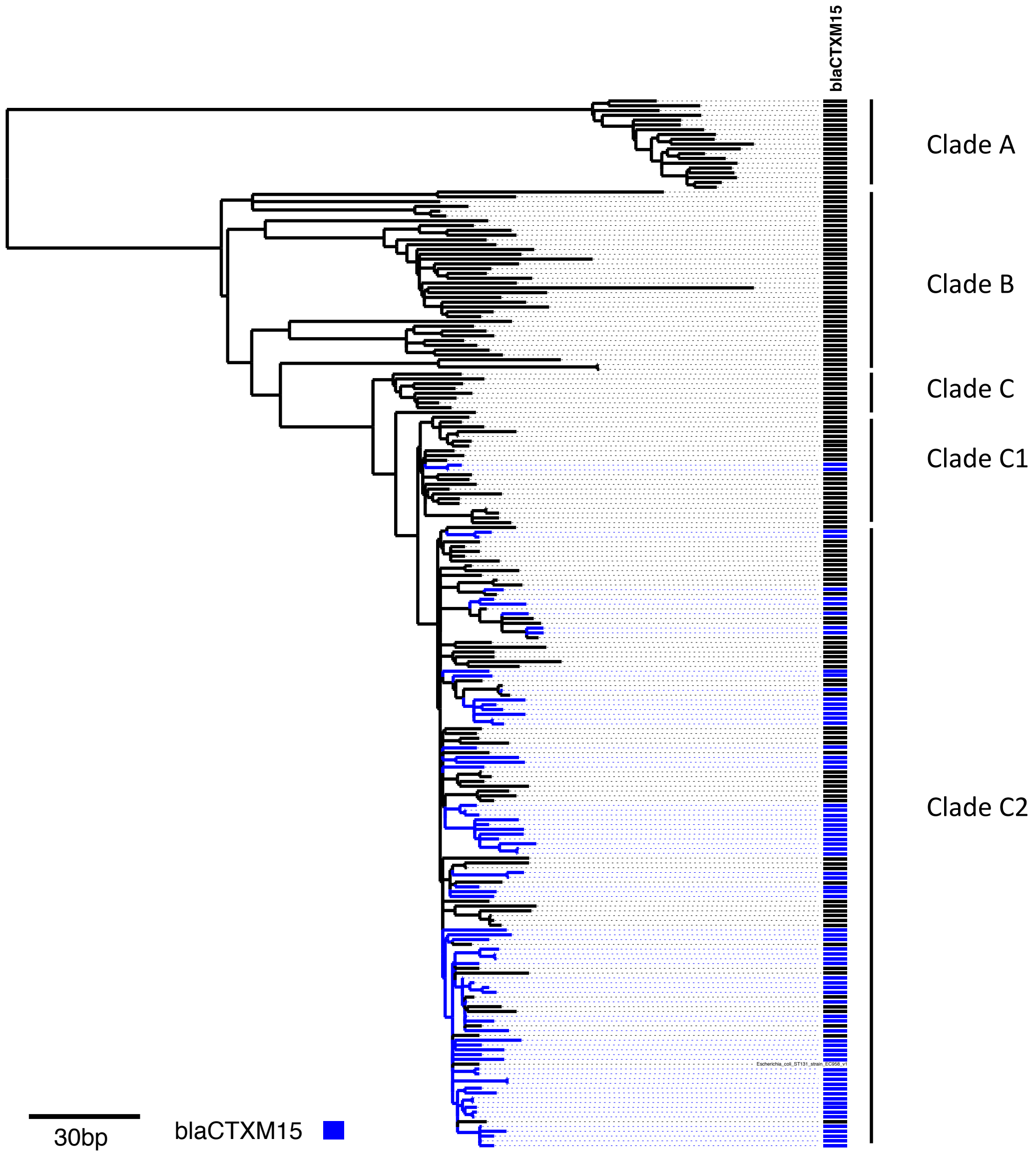

Supplemental Figure S3. Parsimony analysis of $bla_{CTX-M-15}$ in the ST131 phylogeny. Both acctran and deltran models gave the same results. Isolates with detected $bla_{CTX-M-15}$ gene and the corresponding branch in the tree are coloured in blue. ST131 clades are labelled on the right.

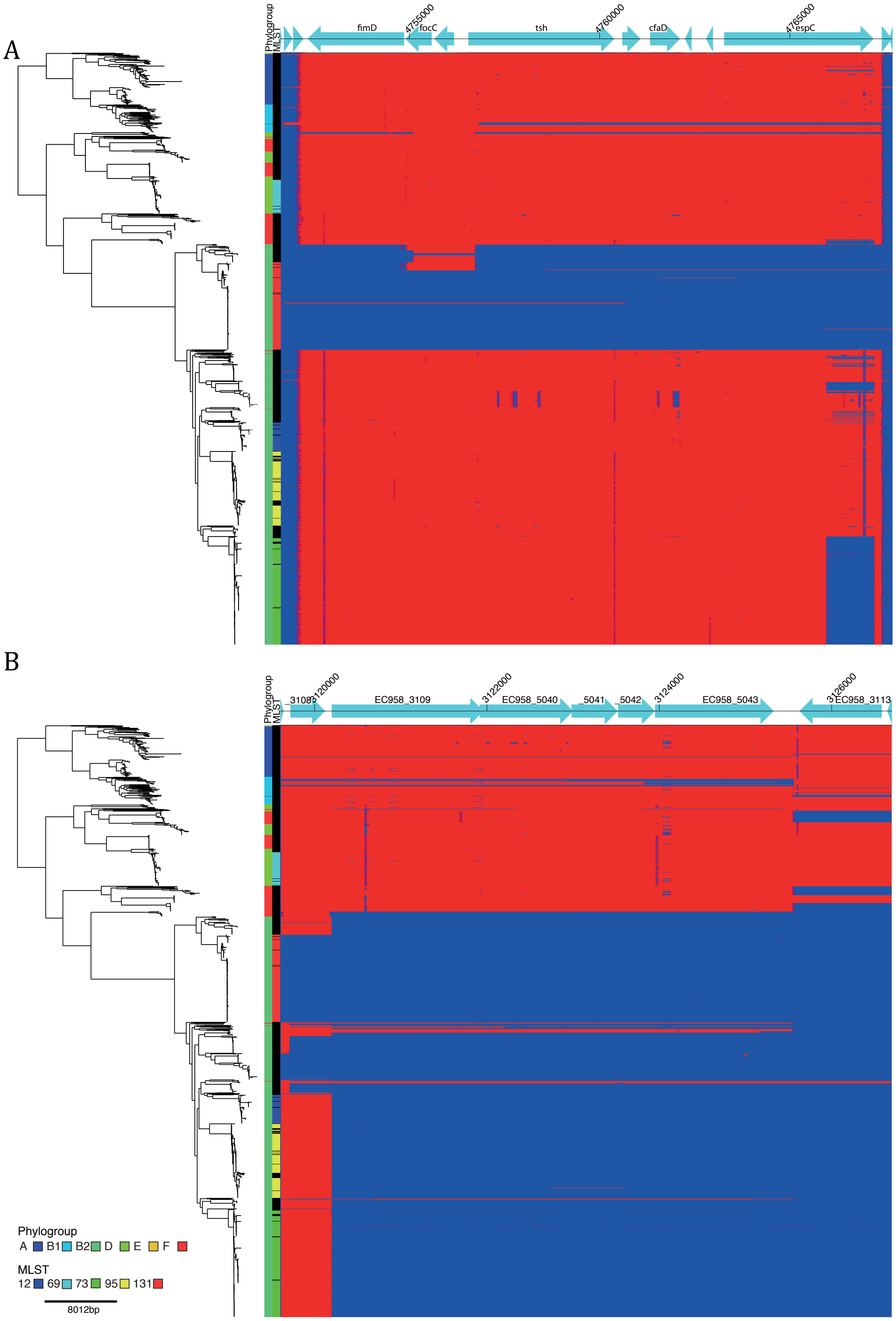

Supplemental Figure S4. Presence of two genomic islands in the maximum-likelihood phylogeny of the bacteraemia-causing *E. coli* population. On the right is shown the phylogroup, ST and mapping of all the reads for each isolate to the reference strain EC958. Blue is presence and red is absence. A) The *espC* island also called ROD3. B) T6SS island (Totsika et al. 2011).

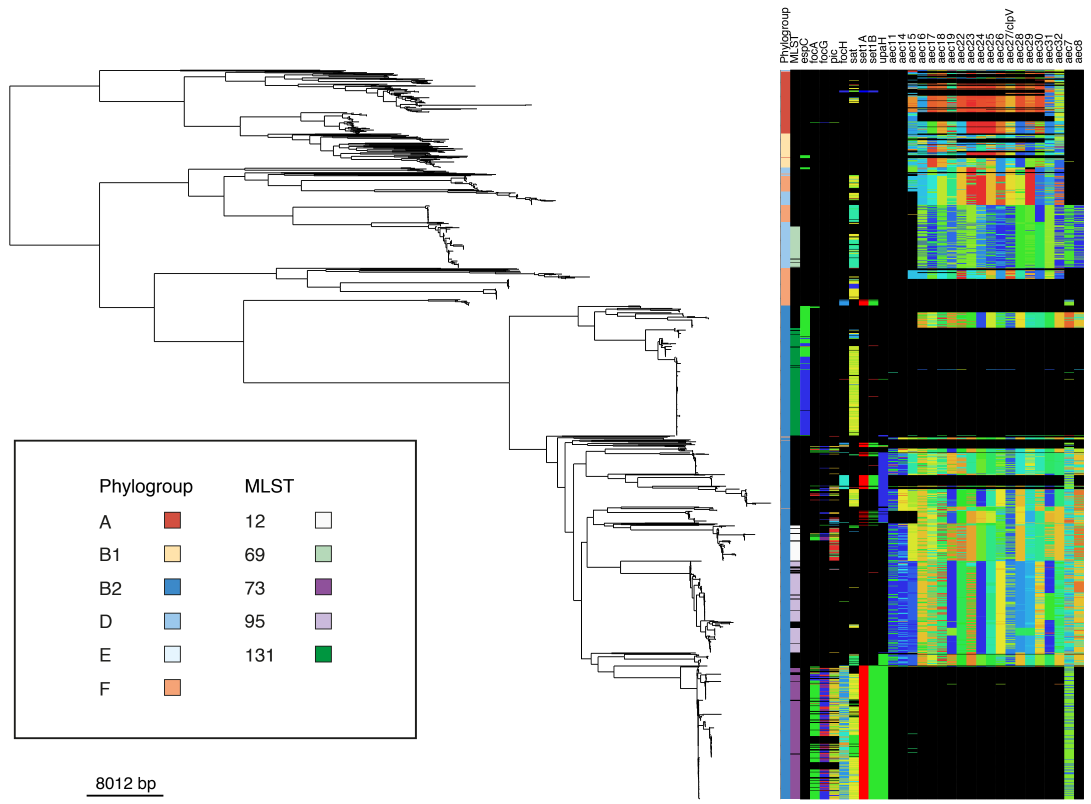

Supplemental Figure S5. Virulence repertoire differences between ST131, ST73 and the rest of the phylogeny. To the right of the phylogenetic tree are shown the phylogroup, five most frequently found sequence types (ST12, ST69, ST73, ST95 and ST131), virulence factor genes that were found to be present in different proportions in ST131, ST73 and the rest of the population (*espC*, *focA*, *focG*, *focH*, *pic*, *sat*, *set1A*, *set1B*, *upaH*, *aec11*, *aec14*, *aec15*, *aec16*, *aec17*, *aec18*, *aec19*, *aec22*, *aec23*, *aec24*, *aec25*, *aec26*, *aec27/clpV*, *aec28*, *aec29*, *aec30*, *aec31*, *aec32*, *aec7*, *aec8*). Different colours in the columns represent different gene types based on clustering the genes in the database at 90% identity and reporting imperfect matches compared to the reference genes as well as genes that may have low coverage for parts of the gene. The phylogenetic tree is the same as in Figure 1.

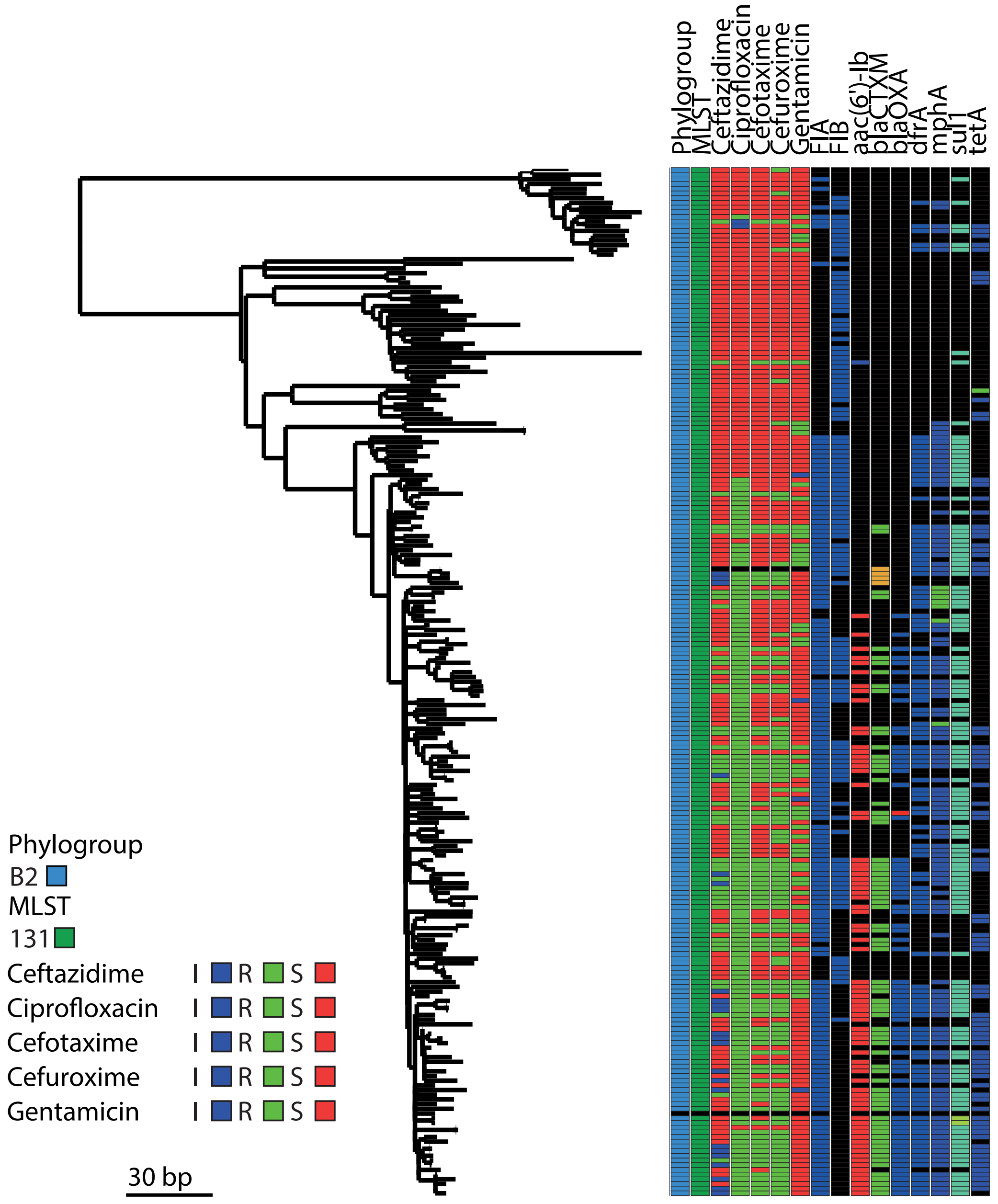

Supplemental Figure S6. Multi-drug resistance plasmids present in ST131. Reference based phylogeny of ST131 with columns to the right representing phylogroup, STs, phenotypic antibiotic resistance data linked to the plasmid (ceftazidime, ciprofloxacin, cefotaxime, cefuroxime and gentamicin), the presence of incFIA and incFIB as well as antibiotic resistance genes carried by the plasmid (*aac(6')-Ib, bla*$_{\text{CTX-M}}$*, bla*$_{\text{OXA}}$*, dfr*A, *mph*A, *sul*1 and *tet*A), black=missing, colour=present. The phylogenetic tree is the same as in Figure 3.

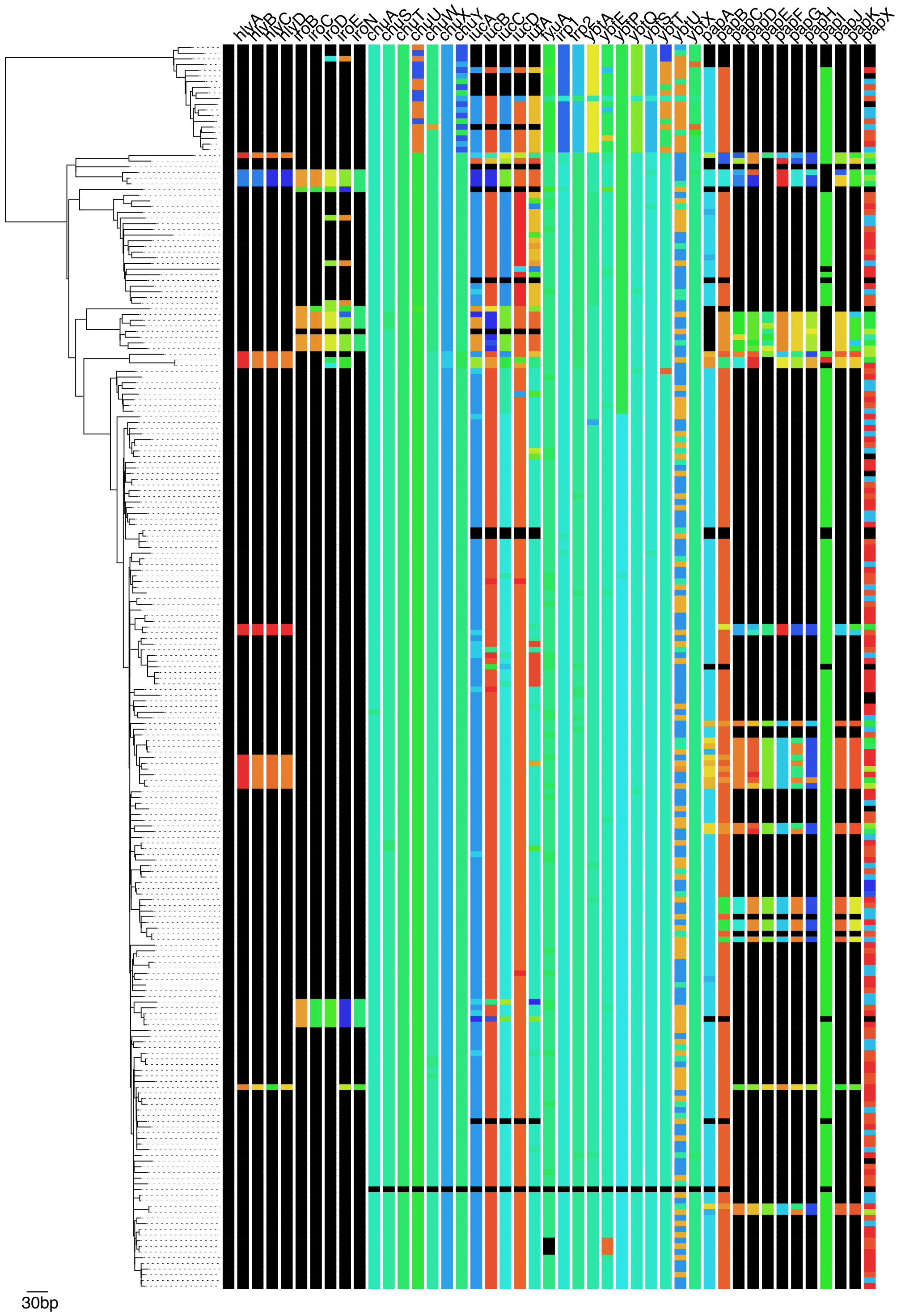

Supplemental Figure S7. ST131 virulence factors. In the figure are presented iron acquisition genes *hly*ABCD, *iro*BCDEN, *chu*ASTUWXY, *iuc*ABCD, *iut*A, *fyu*A, *irp*1, *irp*2, *ybt*AEPQSTUX and P fimbriae genes *pap*ABCDEFGHIJKX. Different colours in the columns represent different gene types based on clustering the genes in the database at 90% identity and reporting imperfect matches compared to the database genes as well as genes that may have low coverage for parts of the gene.

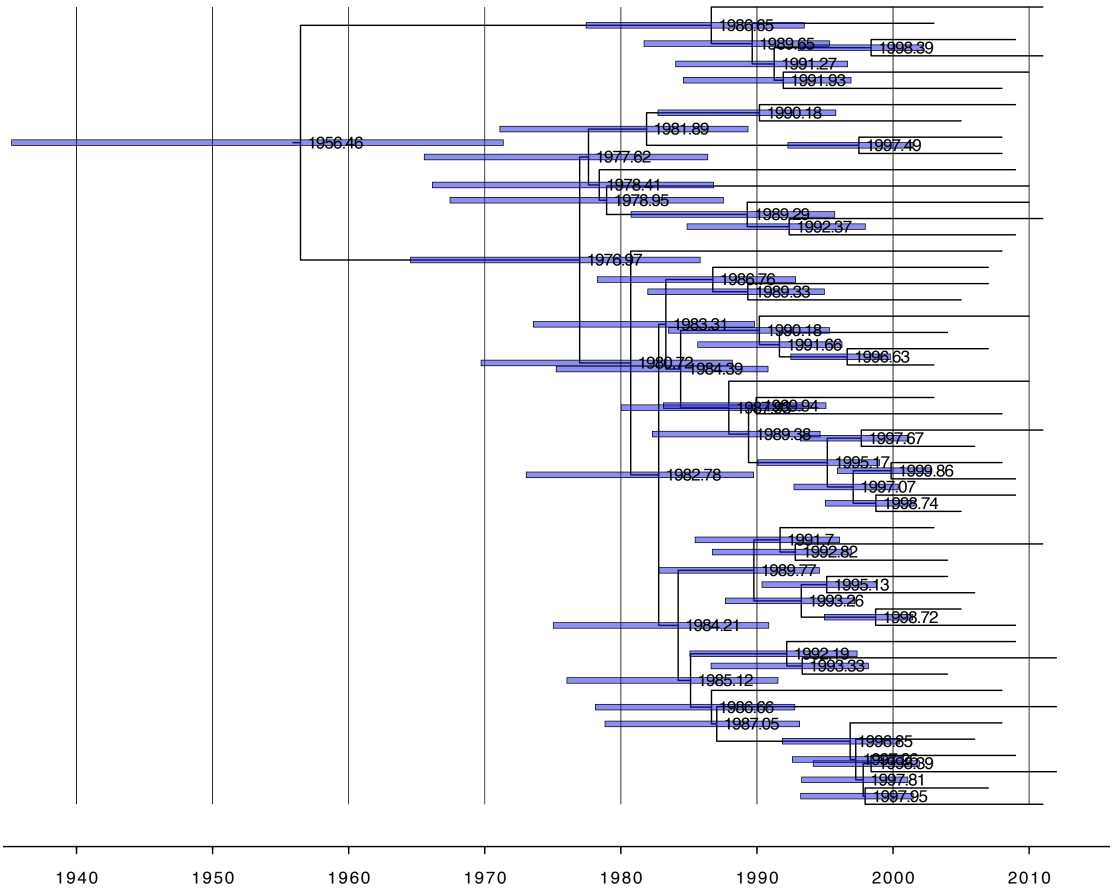

Supplemental Figure S8. Dated tree of the ST69 phylogeny. The divergence times of each node is shown in the figure as well as the 95% highest posterior density (HPD) interval. On the x-axis is time in years. Figure drawn with FigTree (tree.bio.ed.ac.uk/software/figtree/).

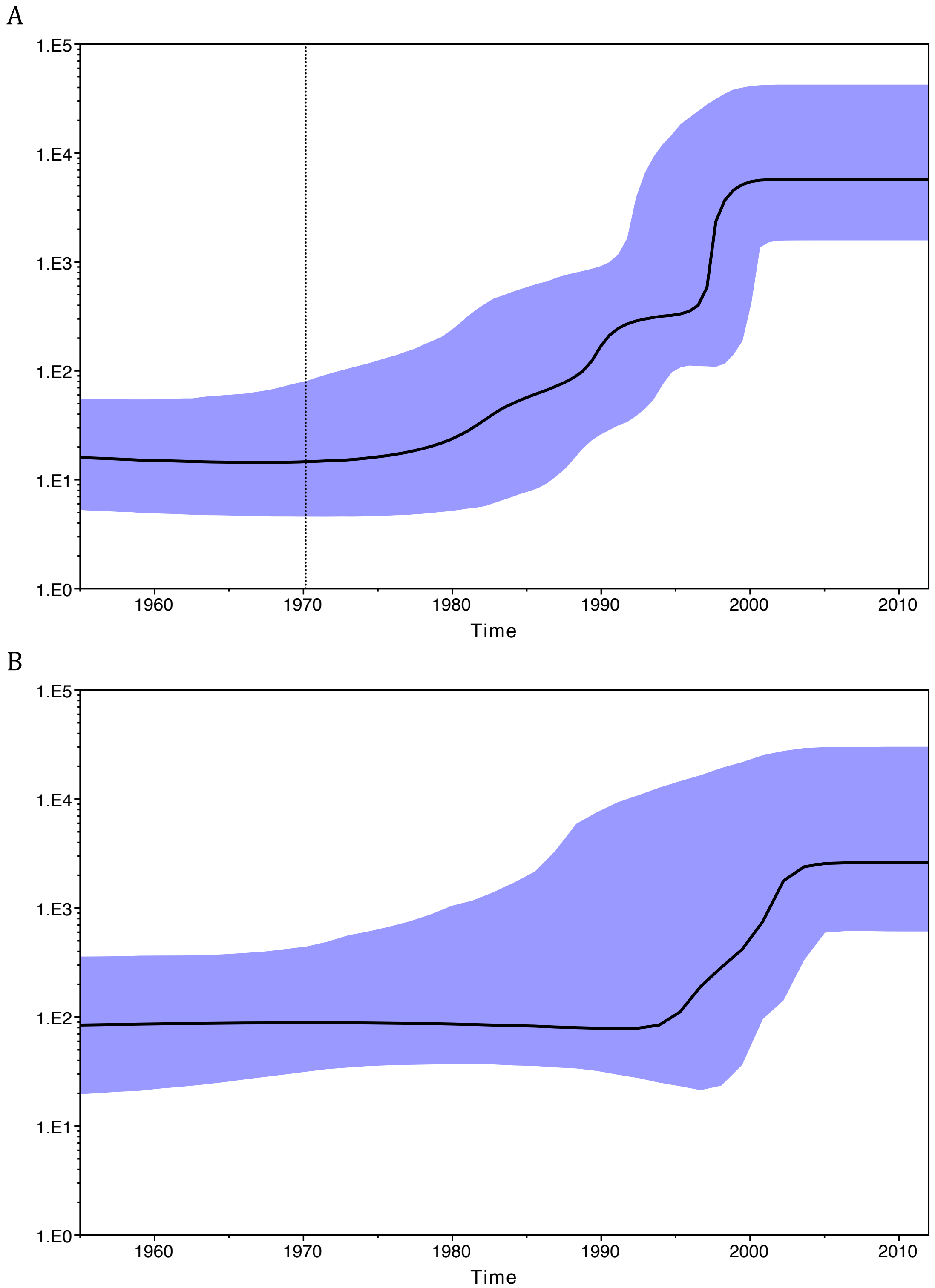

Supplemental Figure S9. Bayesian skyline plot on estimated population size. A) Bayesian Skyline plot of ST69. On the x-axis time (years) and on the y-axis the population size. B) Bayesian Skyline plot of ST131.

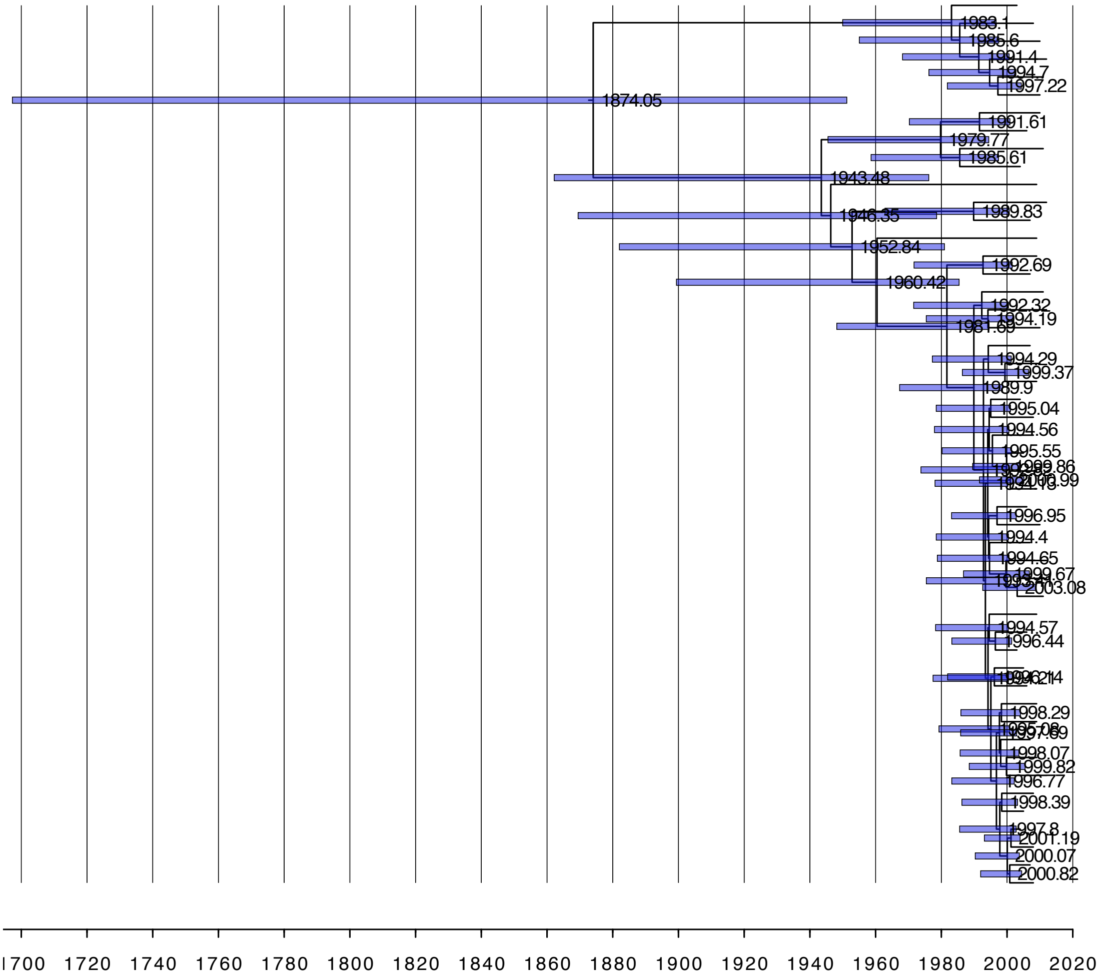

Supplemental Figure S10. The divergence times of each node is shown in the figure as well as the 95% highest posterior density (HPD) interval. On the x-axis is time in years. Figure drawn with FigTree (tree.bio.ed.ac.uk/software/figtree/).

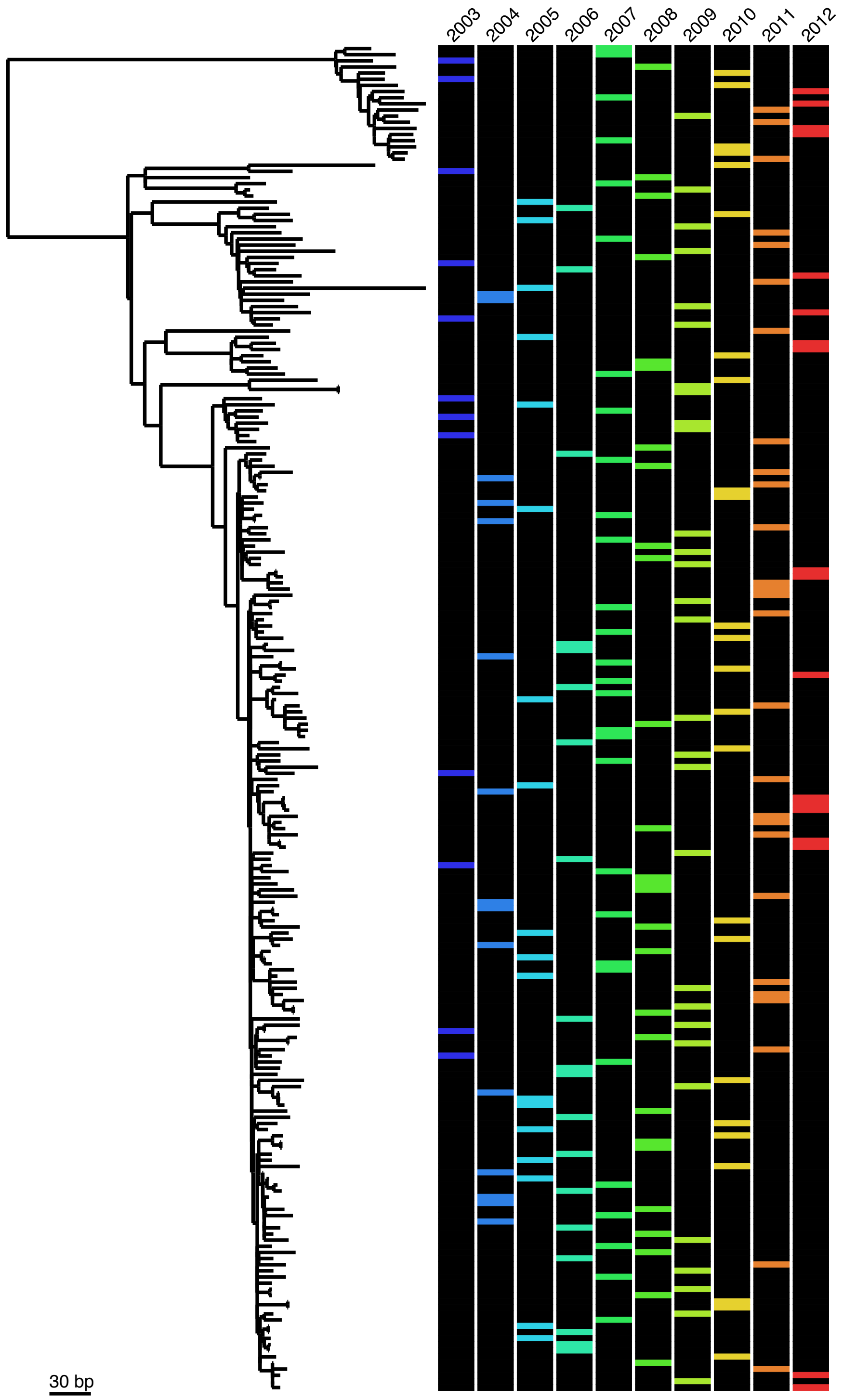

Supplemental Figure S11. ST131 isolation years. Each column presents the isolates from each year from 2003 (blue) to 2012 (red).

References

Ben Zakour NL, Alsheikh-Hussain AS, Ashcroft MM, Khanh Nhu NT, Roberts LW, Stanton-Cook M, Schembri MA, Beatson SA. 2016. Sequential Acquisition of Virulence and Fluoroquinolone Resistance Has Shaped the Evolution of Escherichia coli ST131. *mBio* **7**(2): e00347-00316.

Price LB, Johnson JR, Aziz M, Clabots C, Johnston B, Tchesnokova V, Nordstrom L, Billig M, Chattopadhyay S, Stegger M et al. 2013. The epidemic of extended-spectrum-beta-lactamase-producing Escherichia coli ST131 is driven by a single highly pathogenic subclone, H30-Rx. *mBio* **4**(6): e00377-00313.

Totsika M, Beatson SA, Sarkar S, Phan MD, Petty NK, Bachmann N, Szubert M, Sidjabat HE, Paterson DL, Upton M et al. 2011. Insights into a multidrug resistant Escherichia coli pathogen of the globally disseminated ST131 lineage: genome analysis and virulence mechanisms. *PloS one* **6**(10): e26578.

Ye J, Coulouris G, Zaretskaya I, Cutcutache I, Rozen S, Madden TL. 2012. Primer-BLAST: a tool to design target-specific primers for polymerase chain reaction. *BMC bioinformatics* **13**: 134.
